# Sex‐specific effects of dietary restriction on physiological variables in Japanese quails

**DOI:** 10.1002/ece3.11405

**Published:** 2024-05-23

**Authors:** Gebrehaweria K. Reda, Sawadi F. Ndunguru, Brigitta Csernus, James K. Lugata, Renáta Knop, Csaba Szabó, Levente Czeglédi, Ádám Z. Lendvai

**Affiliations:** ^1^ Department of Animal Science, Institute of Animal Science, Biotechnology and Nature Conservation, Faculty of Agricultural and Food Sciences and Environmental Management University of Debrecen Debrecen Hungary; ^2^ Doctoral School of Animal Science University of Debrecen Debrecen Hungary; ^3^ Department of Evolutionary Zoology and Human Biology, Faculty of Life Science University of Debrecen Debrecen Hungary; ^4^ Department of Animal Nutrition and Physiology, Faculty of Agriculture and Food Sciences and Environmental Management University of Debrecen Debrecen Hungary

**Keywords:** body mass, *Coturnix japonica*, IGF‐1, nutritional limitation, sex difference, triglycerides

## Abstract

Nutritional limitation is a common phenomenon in nature that leads to trade‐offs among processes competing for limited resources. These trade‐offs are mediated by changes in physiological traits such as growth factors and circulating lipids. However, studies addressing the sex‐specific effect of nutritional deficiency on these physiological variables are limited in birds. We used dietary restriction to mimic the depletion of resources to various degrees and investigated sex‐specific effects on circulating levels of insulin‐like growth factor 1 (IGF‐1) and triglycerides in Japanese quails (*Coturnix japonica*) subjected to ad libitum, 20%, 30% or 40% restriction of their daily requirement, for 2 weeks. We also explored the association of both physiological variables with body mass and egg production. While dietary restriction showed no effects on circulating IGF‐1, this hormone exhibited a marked sexual difference, with females having 64.7% higher IGF‐1 levels than males. Dietary restriction significantly reduced plasma triglyceride levels in both sexes. Females showed more than six‐fold higher triglyceride levels than males. Triglyceride levels were positively associated with body mass in females while showed not association in males. Overall, our findings revealed sex‐specific expression of physiological variables under dietary restriction conditions, which coincide with body size.

## Graphical abstract

Trade‐offs among processes competing for limited resources are mediated by changes in physiological traits such as growth factors and circulating lipids. However, studies addressing the sex‐specific effect of nutritional deficiency on these physiological variables are limited in birds. Our findings reveal a sex‐specific expression of physiological variables under dietary restriction conditions and coincide with body size in Japanese quails.

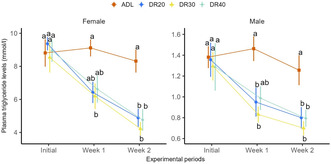

## 1 INTRODUCTION

Nutrition plays an essential role in physiology and affects several vital processes, including growth, development, reproduction and immune functions. A deficiency in whole food or some essential nutrients can profoundly affect an organism's physiology and corresponding fitness (Buchanan et al., 2022; Lee et al., 2008; Magwere et al., 2004). The availability of these essential nutrients in the environment varies considerably, depending on factors such as climate, season and the presence of other organisms (competitors and predators) (Birnie‐Gauvin et al., 2017; Kumar et al., 2022). Consequently, these environmental factors often lead to significant differences in the types and amounts of nutrients available to different species and may have important physiology implications (Jenni‐Eiermann & Jenni, 2012; Lihoreau et al., 2015; McWilliams et al., 2004).

Dietary restriction (DR) is a robust nutritional manipulation that mimics resource limitations. It affects organisms' life‐history traits by influencing physiological and molecular pathways (Cornejo et al., 2007; Du et al., 2023; Kitaysky et al., 2001; Regan et al., 2020). One physiological marker of nutritional availability affecting life history is the insulin‐like growth factor 1 (IGF‐1). IGF‐1 is an essential peptide hormone that exerts pleiotropic effects on cellular growth, proliferation, metabolism and survival in almost all developmental stages. IGF‐1 regulates life‐history traits, essentially, growth, development, reproduction and lifespan across diverse taxa (Lodjak et al., 2018; Lodjak & Verhulst, 2020; Regan et al., 2020). In response to growth hormone and nutritional availability, IGF‐1 is primarily produced in the liver, transported by IGF‐1‐binding globulins and exerts its effects by binding to and activating its tyrosine kinase membrane receptors on cell surfaces. This initiates signal transduction to the intracellular signalling cascades, further activating intracellular kinases, including the mechanistic target of rapamycin (mTOR), which facilitates protein synthesis, cell growth and differentiation (Allard & Duan, 2018; Laron, 2001; Oldham & Hafen, 2003). From an ecological perspective, IGF‐1 is viewed as a regulator of life‐history trade‐offs (Lewin et al., 2017; Lodjak et al., 2018). Variations in IGF‐1 levels among individuals can lead to variations in growth rates and sizes within a population, potentially influencing natural selection and evolutionary processes. For example, individuals with higher IGF‐1 levels may grow larger or mature faster, thereby affecting their survival and reproductive success (Lodjak et al., 2014, 2017; Regan et al., 2020). Differences in IGF‐1 levels among individuals of the same or different species can influence competitive outcomes, especially in contexts where size or growth rates determine access to resources or mates (Oddie, 2000; Ridenour et al., 2023). Within a given species, IGF‐1 levels are positively correlated with body mass, while IGF‐1 deficiency is associated with dwarfism due to reduced growth rates and a slower metabolism (Berryman et al., 2008; Stuart & Page, 2010). Contrarily, across species, IGF‐1 level has been reported to negatively correlate with body mass, which is also proposed to be a function of longevity (Lodjak & Verhulst, 2020; Stuart & Page, 2010).

Studies on model organisms, ranging from yeast to mammals, have revealed that DR decreases insulin/insulin‐like signalling (Bonkowski et al., 2006; Fontana et al., 2010; Klement & Fink, 2016). However, the effect of DR is specific to species, sex, age and mode of intervention (Bacon, 1986; Kewan et al., 2021; Luna‐Castillo et al., 2022; Mellouk et al., 2018). For instance, in mammal models and humans, long‐term DR reduces circulating IGF‐1 levels (Fontana et al., 2008, 2016; Lettieri‐Barbato et al., 2016; Mitchell et al., 2016). However, other studies on rats indicate no significant effect of DR on circulating IGF‐1 (Hanjani et al., 2021) and it also does not alter *IGF1* gene expression in the brain (Marsh et al., 2008) and liver (Masternak et al., 2005) of mice. Furthermore, the effect of DR on plasma IGF‐1 appears to diminish with age in mammals (Breese et al., 1991; Ramsey et al., 2000). Therefore, the nature of the relationship between dietary restriction and IGF‐1 levels is context‐dependent, which needs further study in other model organisms, such as Japanese quails among birds.

Furthermore, energy metabolism and hormonal regulation are markedly different in birds compared to mammals and physiological responses to food restriction may show different patterns. Birds exhibit substantially higher metabolic rates and maintain higher glucose levels, while having more than twice as long lifespans as size‐matched mammals. This observation seems counterintuitive to the general rule that animals with higher metabolic rates usually have shorter lifespans (Holmes et al., 2001; Satoh, 2021). This paradox in birds may arise from their variable physiological response to nutritional availability, which differs from what is observed in mammals. Therefore, conclusions drawn from nutritional physiology studies conducted in mammals cannot be directly applied to avian systems. Additionally, the physiological response to dietary restriction may vary among avian species. For instance, we previously found that DR decreased liver *IGF1* gene expression in male and female Japanese quails (*Coturnix japonica*) (Reda, Ndunguru, Csernus, Knop, Lugata, et al., 2024), whereas other studies have reported that DR increased plasma IGF‐1 in female canaries (*Serinus canaria*) (Hargitai et al., 2022), broiler chicken hens (Hocking et al., 1994) and male and female bearded reedlings (*Panurus biarmicus*) (Tóth et al., 2022). These mixed results indicate that, based on the existing evidence, it is difficult to draw a general conclusion about how changes in food availability affect the IGF‐1 system. Given the paradoxical observations in birds, it becomes essential to investigate the IGF‐1 system across gradients of dietary availability for a comprehensive understanding of the distinct patterns of energy metabolism and hormonal regulation in avian models under laboratory conditions. This is crucial for gaining insights that cannot be extrapolated solely from mammalian studies. Recognising IGF‐1 as a key proxy for growth and reproduction, as well as a regulator of trade‐offs, examining its changes under different levels of food restriction provides valuable insights into how organisms respond to limited nutritional resources and the subsequent impact on their physiological functions. Furthermore, the majority of bird studies have focused on juvenile/nestling stages, overlooking processes in adult subjects. The phenotypic differences between males and females could also contribute to plastic IGF‐1 response to nutritional variation, which calls for further study.

Differences in reproductive investments between the sexes are expected to lead to sex‐specific effects of nutrition on physiology and overall fitness (Maklakov et al., 2008; Vedder et al., 2005). Sex has been shown to have a significant effect on plasma IGF‐1 levels across different bird species and growth stages (Bacon et al., 1993; Ballard et al., 1990; McMurtry et al., 1997; Tóth et al., 2018, 2022). Several factors, including gonadal steroids, feeding behaviour, energy metabolism and growth rate, modulate IGF‐1 production and may contribute to sex differences (Cleveland & Weber, 2015; Liu et al., 2016; Meinhardt & Ho, 2006).

Another critical physiological marker of nutritional status is the level of circulating triglycerides. Ecologically, they have been proposed as the most reliable indicators of nutritional state (Masello & Quillfeldt, 2004; Peres et al., 2014). The concentration of triglycerides reflects an organism's accessibility to food sources in its environment. For instance, species with a high intake of fatty foods may exhibit elevated plasma triglyceride levels, suggesting adaptation to a particular nutritional niche (Jackson et al., 2023; Smith et al., 2021). Triglyceride levels have a strong correlation with body mass and predict reproductive success (Jenni‐Eiermann et al., 2002; Masello & Quillfeldt, 2004). Triglycerides are excellent indicators of fat metabolism in birds, as they function as primary energy reserves (Roccio et al., 2021; Smith & McWilliams, 2009). The levels of triglycerides in their blood are influenced by nutrient availability (Fokidis et al., 2012). Dietary restriction has been shown to reduce triglyceride levels (Kudchodkar et al., 1977; Solon‐Biet et al., 2015; Zhu et al., 2004), which is a sign of increased use of energy reserves. In mammals, dietary restriction elicits conflicting effects on triglyceride levels: restricted males show lower, while females exhibit higher levels than their unrestricted counterparts (Mattison et al., 2012). However, the impact of varying levels of dietary restriction and sex on circulating triglycerides in birds remains unknown.

Therefore, the present study aims to investigate sex‐specific regulation of circulating levels of IGF‐1 and triglyceride in adult Japanese quails exposed to varying degrees of dietary restriction. Japanese quails are sexually size‐dimorphic, with *females being larger than males* (Okuno et al., 2020). We hypothesise that plasma levels of IGF‐1 and triglycerides exhibit a negative response to dietary restriction, with the magnitude of this response corresponding with the degree of restriction. Furthermore, we anticipated that variations in sexual size dimorphism would be reflected in differences in the IGF‐1 and triglyceride levels in response to the gradient of dietary restriction.

## 2 MATERIALS AND METHODS

### 2.1 Experimental animals

We used Japanese quails as an experimental model. The Japanese quail is a well‐known avian model for studying molecular, physiological and phenotypic fitness in response to environmental cues. The domesticated Japanese quail is heterogeneous, retaining several wild‐type characteristics, such as smaller body size compared to other domesticated species and robustness in both health and reproduction. They have quick generational turnover and early sexual maturity, which make them crucial for research purposes (Ball & Balthazart, 2010; Ottinger, 2007; Ottinger et al., 2005). Japanese quails are size‐dimorphic birds, with adult females being heavier than males. Consequently, females take longer than males to reach sexual maturity. For the first 5 weeks, both males and females grow at a similar pace. However, after this period, males accelerate their growth and typically reach sexual maturity 1 or 2 weeks earlier than females, usually at 5 to 6 weeks of age, while females reach sexual maturity at 6 to 7 weeks of age (Cahyadi et al., 2019; Hassan et al., 2003; Huss et al., 2008; Ottinger et al., 2005). Under al libitum feeding conditions, hens weekly lay 5–7 eggs (Mahrose et al., 2022; Reda, Ndunguru, Csernus, Knop, Szabó, et al., 2024). Wild Japanese quails are ground‐nesting birds that inhabit grassy fields, croplands, mountain slopes and meadow habitats. They feed on grass and crop seeds, various types of vegetation and occasionally small insects and generally have a lifespan of 2–3 years (Baer et al., 2015; Ottinger, 2001).

### 2.2 Housing and feeding

We obtained four‐week‐old Japanese quail (*Coturnix japonica*) chicks from a commercial quail breeder (Budai Fürjészet, Hungary) and housed them in the Animal House of the Institute of Animal Science, Biotechnology and Nature Conservation of the University of Debrecen (Hungary). Birds were kept for four more weeks until maturity. We then selected 64 birds comprising both sexes with similar sex‐specific body mass (average mass of females: 275.3 ± 3.64 g; males: 238.79 ± 2.92 g) and housed them in individual cages for 7 acclimation days on ad libitum feed and free access to water. The experimental room was maintained at 25 ± 3°C and 60%–75% relative humidity. The light schedule was fixed at 12:12 light:dark cycle using lighting timers.

The basal quail feed was formulated on a corn, wheat and soybean meal basis corresponding to the nutrient requirement of breeder quails (Table A1 in Appendix 2) (National Research Council, 1994). During the 7‐day acclimation period, we provided ad libitum feed daily in the morning and measured the feed intake of each individual with a 24‐h interval following the previous feeding. The average daily feed intake was calculated as the mean of the seven measurements of individual birds. The average feed intake during the acclimation period was 29.67 ± 3.73 g for female and 20.51 ± 4.32 g for male birds. Additionally, we recorded each bird's body mass at the beginning and end of the acclimation period to analyse any potential change in mass. Both body mass and feed intake remained stable before the onset of the experiment (body mass: *p* = .615; intake: *p* = .384).

### 2.3 Experimental design

The experiment was designed as a 2 (sex) × 4 (DR) factorial design, in which 32 female and 32 male birds (8 birds per treatment) were randomly assigned to one of four dietary treatments. Birds were fed diets consisting of 80% (DR20), 70% (DR30) and 60% (DR40) of their individual average feed intake, while the control group was fed ad libitum (ADL). The birds were classified into eight experimental blocks based on the vertical position of their cage in the system's staircase, where both sex and all treatments were placed in a block. The experiment was conducted over 2 weeks from 20 July to 6 August 2021. Daily feed intake was measured and analysed to monitor potential significant changes in the temporal intake of the control birds. However, no significant variations in feed intake (*p* = .402) were observed throughout the experimental period in controls, suggesting no shift in food requirement or physiology.

### 2.4 Blood sampling

Immediately after lights on in the morning, we removed all the feeders to maintain similar feeding conditions (empty gut) between the ADL and restricted birds during sampling points. Blood samples were collected at the beginning, middle and the last day of the experimental period (days 0, 7 and 14 of the trial). To ease the blood sampling procedure and reducing the overlap between concurrent bleedings, we staggered the beginning of the experiment and blood sampling so that samples from only 16 birds (2 birds from each treatment group and sex) were collected each day. There was no staggering time effect on IGF‐1 (*p* = .450), triglyceride levels (*p* = .489) and body mass (*p* = .469). To minimise handling time, on each sampling day, several people simultaneously bled 2 to 3 birds (median time 155.5 s, range 78–435 s from the time when the door of the experiment room opened). There was no significant handling‐time‐induced variation in IGF‐1 (*p* = .495) and triglyceride levels (*p* = .750). After the first round of blood collection, we left the experimental room undisturbed for an hour and started another session of blood collection on the remaining birds in the same manner. There was no session effect on either IGF‐1 (*p* = .694) or triglycerides (*p* = .607) levels. We conducted blood sampling from 8:00 to 10:00 a.m., followed by feeding the birds and cleaning their enclosures. Subsequently, the birds were left undisturbed for the remainder of the day. The blood samples were collected from the brachial vein following venepuncture into heparinised capillary tubes, then transferred the blood into 0.5 mL microcentrifuge tubes. The collected blood samples were immediately centrifuged at 9000 *g* for 10 min and the separated plasma was stored at −80°C until further laboratory use (30 September to 10 October 2021).

### 2.5 IGF‐1 enzyme‐linked immunosorbent assay (ELISA)

We measured plasma IGF‐1 levels in duplicates using a competitive ELISA assay procedure described earlier (Mahr et al., 2020). Briefly, we coated Nunc™ 96‐Well Polypropylene MicroWell™ microtiter plates (Thermo Scientific™) overnight using 100 μL polyclonal anti‐serum. The next day, before adding the standards and samples into the wells, we removed the unbounded capture antibody through forceful expulsion and washed using 250 μL washing buffer in each well. We diluted 11 μL plasma samples with 9 μL PBS in the wells. Then, we pipetted 100 μL biotinylated IGF‐1 tracer solution into each well and incubated for 2 h at room temperature. After incubation and washing, we pipetted 100 μL streptavidin‐horseradish peroxidase (HRP) enzyme conjugate dilution into each well and incubated for 30 min. After washing the HRP for removal of the unbound enzyme, we added 100 μL 3,3′,5,5′‐Tetramethylbenzidine (TMB) chromogenic substrate (Acros Organics) to each well and incubated at room temperature for 30 min. We stopped the enzymatic reaction by adding 100 μL of 1 M H_2_SO_4_ solution into each well and measured absorbance at 450 nm (with reference at 620 nm) using a Magellan F50 spectrophotometer plate reader (Fornax Technologies GmbH, Bühlinger Straße 56, Germany). Coefficients of variation for intra‐ and inter‐assay were 4.16% and 8.39%, respectively.

### 2.6 Triglyceride measurements

We analysed plasma triglyceride levels in duplicates using a photometric method with a half‐automatic analyser (Lab‐Analyse, Orvostechnika Ltd., Budapest, Hungary) according to the manufacturer's protocol. Briefly, we added 5 μL plasma sample into a unit of triglyceride reagent (pH = 7.20 of 50 mmol/L PIPES buffer, 5 mmol/L Mg++, 4 mmol/L 4‐chlorophenol, 2.0 mmol/L ATP, 2000 U/L lipoprotein lipase, 400 U/L glycerol kinase, 1500 U/L glycerol‐3‐phosphate oxidase (GPO), 2000 U/L peroxidase and 0.4 mmol/L 4‐aminoantipyrine). We added the sample to the reagent, thoroughly mixed and incubated it for 5 min, then measured it in the photometric device at a wavelength of 505 nm. We used the triglyceride reagent as a blank before measurement.

### 2.7 Ethical declaration

The experiment was approved by the Ethical Committee for Animal Use of the University of Debrecen, Hungary (Protocol No. 5/2021/DEMAB) and followed all institutional and national regulations.

### 2.8 Statistical analyses

All data analyses were performed using R v. 4.2.2 ‘Innocent and Trusting’ (R Core Team, 2022). We employed a stepwise backward model selection procedure for model fitting (Zhang, 2016). We utilised the ‘lme4’ package (Bates et al., 2015) and version 3.1‐3 of the ‘lmerTest’ package (Kuznetsova et al., 2017) to fit the models and calculate *p*‐values. We used the Tukey test as a post hoc test with *p* < .05 significance level for mean comparison and bars set as means ± SEM.

To test the effect of treatments, restriction periods (weeks) and sex on plasma IGF‐1 and triglyceride levels and mass loss, we employed linear mixed‐effects models. The structure of the initial saturated models included treatment, restriction weeks and sex as fixed factors, along with all their possible interactions, while individual bird identity nested in the experimental block was treated as a random intercept. We controlled for the effect of individual body mass variation by employing within‐individual centring. However, as this effect was not significant, we excluded it from the model. We transformed the physiological variables using the natural logarithm. Therefore, the final model for mass loss analyses was: *lmer(mass loss~treatment × (week + sex) + week × sex + (1|block/birdID))*. We excluded the three‐way interaction of the predictor variables since it does not have a significant contribution to the models predictive power. Mass losses were calculated by subtracting the initial body mass from the mass recorded in week 1 and week 2. None of the fixed variables, except for sex, contributed to the variability of IGF‐1. Therefore, the final model contains only sex. The final model for triglyceride levels analyses was as follow: *lmer(log(triglyceride)~treatment × week + sex + (1|block/birdID))*.

We also utilised body mass and egg production data from the same experiment reported previously (Reda, Ndunguru, Csernus, Knop, Szabó, et al., 2024), to analyse associations with plasma IGF‐1 and triglyceride levels. To match the daily egg parameters and physiological variables across time points (day 0, 7, 14), we summarised the number of eggs laid from day 1 to day 7 as the egg number for week 1 and from day 8 to day 14 as the egg number for week 2. Additionally, the egg mass on day 0 is considered the initial egg mass; the average egg mass from day 1 to 7 represents the egg mass for week 1 and the average egg mass from day 8 to 14 represents the egg mass for week 2. Therefore, we fitted models to test how body mass, egg mass and egg number explain variations in IGF‐1 and triglycerides levels. We employed within‐treatment centring for body mass, egg number and egg mass (subtracting treatment mean from each individual measurement) to disentangle the treatment‐induced and the residual variation in these factors (Van de Pol & Wright, 2009). In the model specifications, we denote these variables by *wt_trait* and *bt_trait*, where *wt* refers to the within‐treatment centred and *bt* to the between‐treatment centred trait (such as mass, egg number or egg mass), respectively. Therefore, to examine the relationship between IGF‐1 and body mass, we employed a linear mixed‐effect model while controlling for effect of sex, week and treatment as fixed and bird identity as random factors. The final model was: *lmer(log(IGF‐1)~wt_mass + bt_mass + sex + (1|birdID))*. We excluded the block since the variance is zero. Additionally, we explored the association between IGF‐1 and egg parameters using a linear mixed‐effect model. To account for between‐treatment variation and weeks, we included a between‐treatment‐centred effect and weeks as a control variable. The final model for the association with egg traits were: *lmer(log(IGF‐1)~wt_eggtrait + bt_eggtrait + week + (1|block/birdID))*, where *eggtrait* corresponds to egg mass or egg number.

Furthermore, we investigated the relationship between plasma triglyceride levels and body mass using a linear mixed‐effect model. This analysis controlled for between‐treatment‐centred body mass, sex, week and interaction between‐within‐treatment centred body mass and sex to account for potential confounding effects. The final model was: *lmer(log(triglyceride)~wt_mass × sex + bt_mass + week + (1|block/birdID))*. Using the same selection method, we have excluded all interactions except the interaction between within‐treatment centred body mass and sex. Similarly, we used a linear mixed‐effect model to analyse the relationship between triglyceride levels and egg parameters. We included between‐treatment centred effects and week as control variables to address between‐treatment variations and effect of restriction time. The final models were as follows: *lmer(log(triglyceride)~wt_eggtraits + bt_eggtraits + week + (1|block/birdID))*, where ‘*eggtraits’* correspond to egg mass and egg number. Finally, we assessed the relationship between plasma IGF‐1 and triglyceride levels using a linear mixed‐effect model while controlling for sex and restriction time to mitigate any sex‐derived influence on the relationship. The final model was: *lmer(log(IGF‐1)~wt_triglyceride × week + bt_triglyceride + sex + (1|birdID))*.

We assessed IGF‐1 across different individuals, using linear mixed‐effect models to partition the variance observed in IGF‐1 levels into components attributable to individual differences. Therefore, variance components were extracted using the ‘VarCorr’ function from ‘lme4’ package. The repeatability was calculated as the ratio of between‐individual variance to the total observed variance.

## 3 RESULTS

### 3.1 Dietary restriction induced significant body mass loss

**FIGURE 1:**
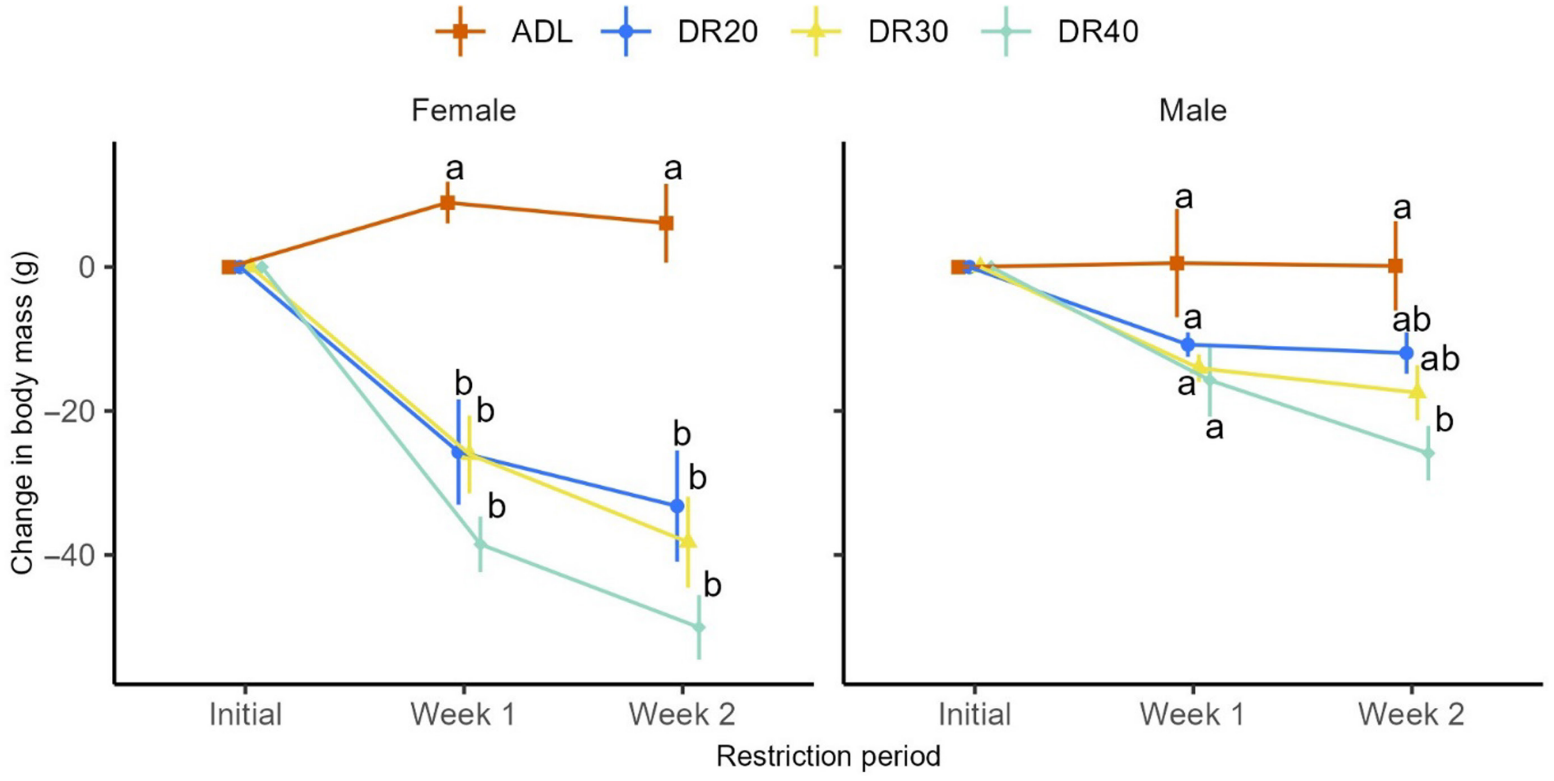
Change in body mass due to dietary restriction across the time points in female and male Japanese quails. Data are calculated by subtracting initial body mass from body mass recoded at the end of week 1or week 2 of the treatment period. Data are represented by the mean ± SEM and were statistically analysed from the linear mixed‐effect model (lmer(mass lost~treatment*(week + sex) + week*sex + (1|block/birdID))). All interaction effects were significant except the interaction among treatment × week × sex (*p* = .328). Means followed by a common letter vertically, on the same time point, are not significantly different (*p* > .05). ADL, ad libitum; DR20, 20% restriction; DR30, 30% restriction; DR40, 40% restriction.

Output of the linear mixed‐effect model showed that dietary restriction induced a loss of body mass in both sexes (treatment: *F*_3,49_ = 19.49, *p* < .001; week: *F*_1,56_ = 34.84, *p* < .001; sex: *F*_1,49_ = 10.58, *p* = .002; treatment × week interaction: *F*_3,56_ = 3.84, *p* = .014; treatment × sex interaction: *F*_3,49_ = 3.44, *p* = .024; week × sex interaction: *F*_1,56_ = 7.05, *p* = .010; Figure 1; Table A2 in Appendix 2). However, this effect was intensive in females. In females, birds in all restriction levels lost body mass compared to controls at both week 1 and week 2. In males, DR20 and DR30 did not induce a significant body mass loss at all‐time points, while the severe restriction group (DR40) showed significant mass loss at the second week of restriction period (*p* = .003; Figure 1, Table A3 in Appendix 2).

### 3.2 Plasma IGF‐1 showed sex‐specific variation

**FIGURE 2:**
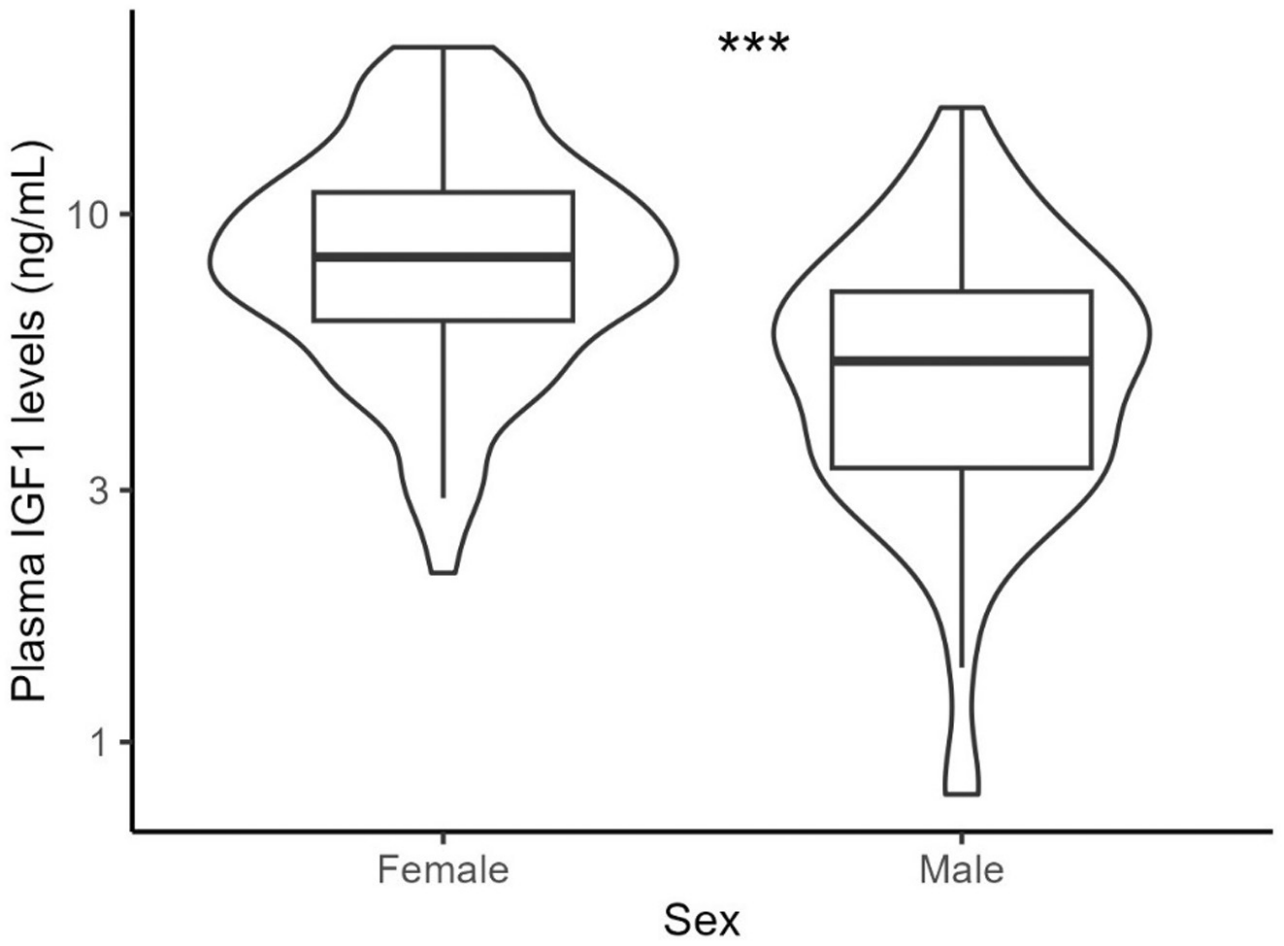
Sex has significant effect on plasma IGF‐1 levels. Data were analysed from 32 birds per sex group in three time points and statistical comparisons were derived from a linear model (*lm(IGF‐1~sex)*). Data were collected on day 0 (initial), week 1 and week 2 periods of the experiment.

**FIGURE A1:**
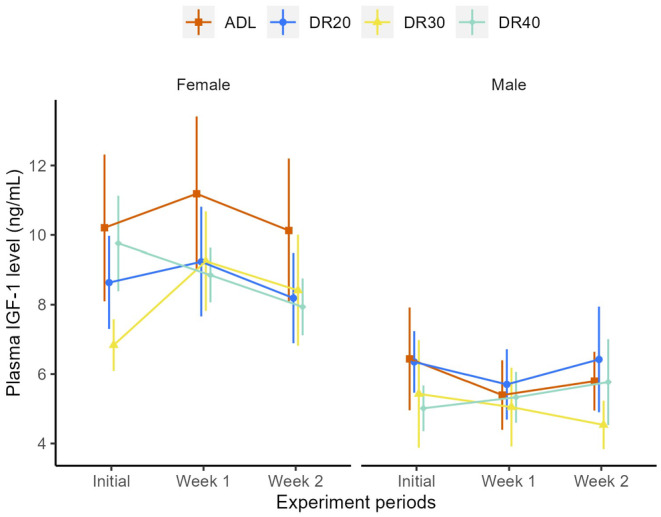
The effect of different dietary restriction regimens on plasma IGF‐1 levels of female and male quails at different time points. Data are represented by the mean ± SEM from eight birds per group and were statistically analysed from the linear mixed effect model (lmer(IGF‐1~treatment*week*sex + (1|block/birdID))). The treatment and experimental period showed no significant effect on IGF‐1 levels. ADL, ad libitum; DR20, 20% restriction; DR30, 30% restriction; DR40, 40% restriction; ns, none significant.

**FIGURE A2:**
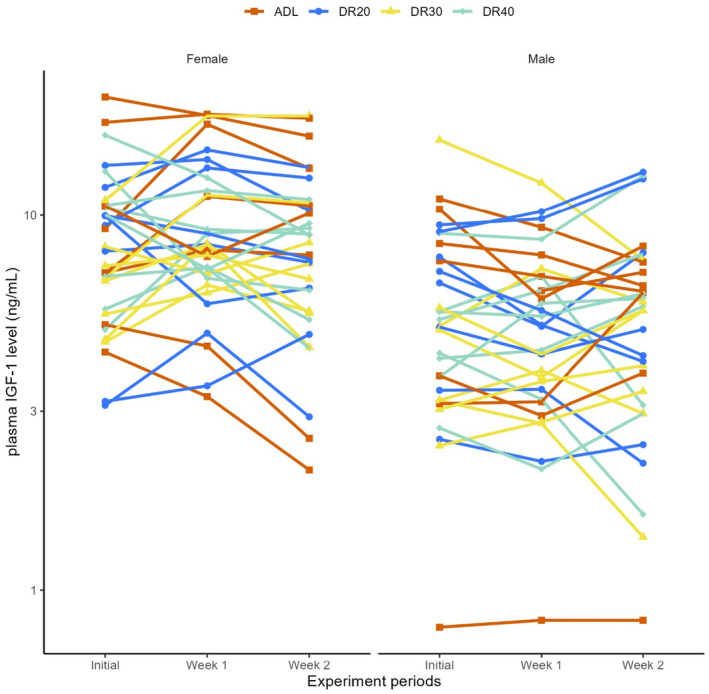
Individuals showed significantly repeatable variable patterns of plasma IGF‐1 levels across two 2‐week restriction period. ADL, ad libitum; DR20, 20% restriction; DR30, 30% restriction; DR40, 40% restriction.

**FIGURE A3:**
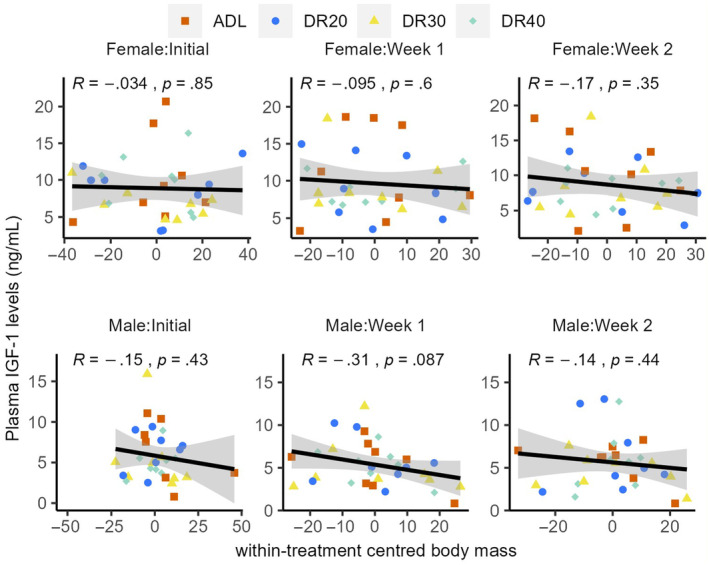
Relationship between body mass and plasma IGF‐1 levels in females (top panels) and males (bottom panels) at three time points of the experiment. The association of plasma IGF‐1 with body mass showed a tendency to be significant only during week 1 in males. ADL, ad libitum; DR20, 20% restriction; DR30, 30% restriction; DR40, 40% restriction.

**FIGURE A4:**
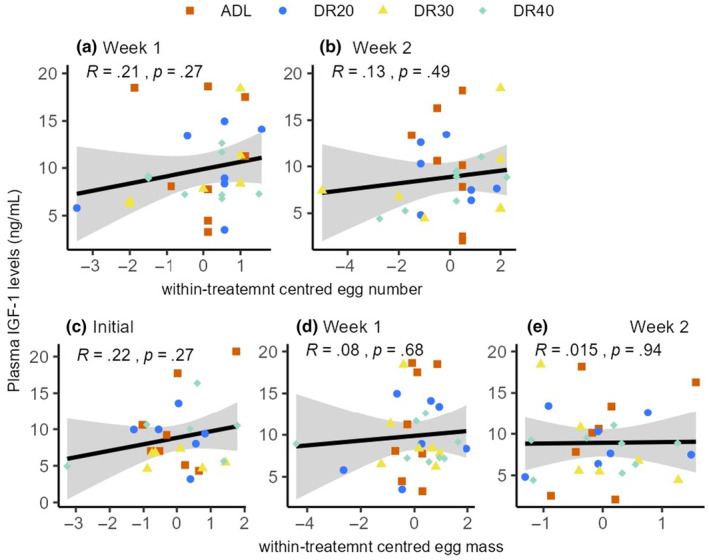
Association of plasma IGF‐1 to egg parameters. Plasma IGF‐1 is not related to (a, b) egg number at week 1 and week 2, (c) initial egg mass and (d, e) egg mass at week 1 and 2. Egg parameters are within‐treatment centred. ADL, ad libitum; DR20, 20% restriction; DR30, 30% restriction; DR40, 40% restriction.

Dietary restriction and its interaction with sex and the restriction period (week) did not affect plasma IGF‐1 levels (treatment: *F*_3,49_ = 0.41, *p* = .743; treatment × week interaction: *F*_6,111.19_ = 0.88, *p* = .511; treatment × sex interaction: *F*_3,49_ = 0.13, *p* = .944; treatment × week × sex interaction: *F*_6,111.19_ = 1.53, *p* = .174; Figure A1 in Appendix 1). Additionally, IGF‐1 levels remained unchanged throughout the restriction periods in both males and females (week: *F*_2,111.19_ = 1.76, *p* = .177; week × sex interaction: *F*_2,111.19_ = 2.02, *p* = .137, Figure A1 in Appendix 1). Females exhibited higher plasma IGF‐1 levels (64.7%) than males at all restriction levels (sex: *F*_1,49_ = 15.67, *p* < .001, Figure 2). Our data also further revealed repeatable individual variation in IGF‐1 levels across the restriction period with high repeatability (repeatability: 0.81, Figure A2 in Appendix 1). Within‐treatment variation in body mass had no significant association with IGF‐1 levels (*p* = .539; Figure A3 in Appendix 1; Table A4 in Appendix 2). IGF‐1 levels were also not associated with individual‐centred body mass (*p* = .421). Additionally, the egg mass and the number of eggs showed no significant relationship with IGF‐1 levels (egg mass: *p* = .579; egg number: *p* = .523; Figure A4 in Appendix 1; Tables A5 and A6 in Appendix 2).

### 3.3 Dietary restriction reduced plasma triglyceride levels

**FIGURE 3:**
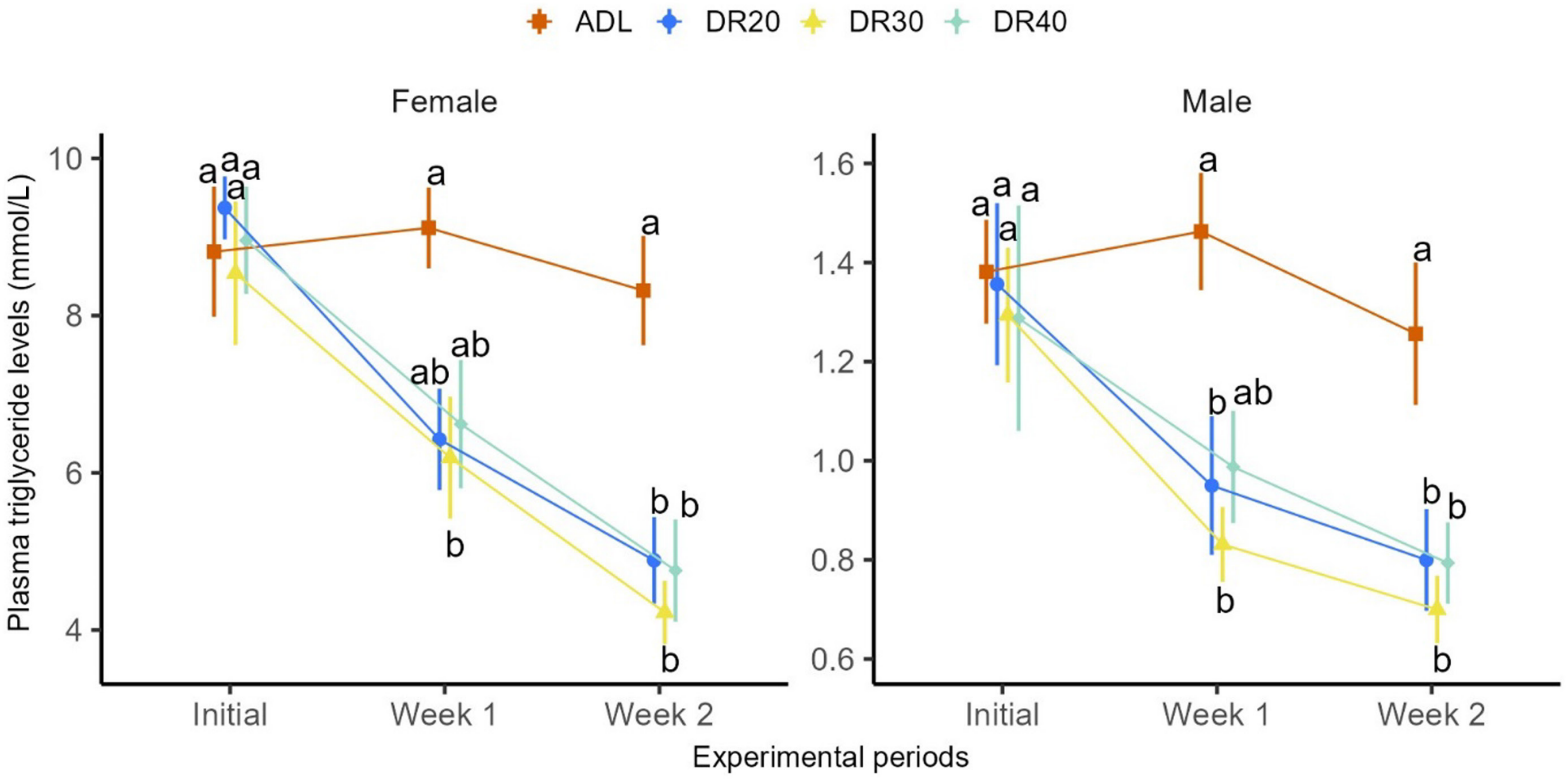
The effect of dietary restriction gradient on plasma triglyceride levels of female and male quails at different time points. Data are represented by the mean ± SEM and were statistically analysed from the linear mixed‐effect model lmer(log(trig)~treatment*week + sex + (1|block/birdID)). Note the different scales for the sexes, reflecting the significant disparity in female and male triglyceride values. The treatment × week interaction was significant (*p* = .001). Means followed by a common letter vertically on the same time point are not significantly different (*p* > .05). ADL, ad libitum; DR20, 20% restriction; DR30, 30% restriction; DR40, 40% restriction.

The final linear mixed‐effect model revealed a significant treatment and treatment by restriction period interaction effect (treatment: *F*_3,52_ = 11.45, *p* < .001; treatment × week: *F*_6,120_ = 4.10, *p* = .001 Figure 3; Table A7 in Appendix 2). Comparing treatment groups at different time points, all the restricted female groups showed significantly lower triglyceride levels than the control group in week 2 (DR20: *p* = .002; DR30: *p* < .001; DR40: *p* = .001, Figure 3; Table A8 in Appendix 2). However, in the first treatment week, DR20 and DR40 treatments imposed marginal reduction in the levels of plasma triglycerides (DR20: *p* = .061, DR40: *p* = .071; Figure 3; Table A8 in Appendix 2), while DR30 treatment showed significant reduction compared to ADL fed group (*p* = .020; Table A8 in Appendix 2). In males, all restricted groups showed significantly lower plasma triglyceride levels than the control group in week 1 (DR20: *p* = .023; DR30: *p* = .006; DR40: *p* = .068, Figure 3; Table A8 in Appendix 2) and week 2 (DR20: *p* = .044; DR30: *p* = .006; DR40: *p* = .043, Figure 3; Table A8 in Appendix 2).

Secondly, we tested the effect of restriction time and found that in both females and males, all restricted groups showed reduced value compared to their baseline plasma triglyceride levels at both week 1 and week 2 (*F*_2,112_ = 24.63, *p* < .001; Figure 3; Table A9 in Appendix 2) except DR40 (Figure 3; Table A9 in Appendix 2). In case of the trend from week 1 to week 2, DR30 and DR40 of female groups showed a significant reduction in week 2 (DR30: *p* = .027; DR40: *p* = .017, Table A9 in Appendix 2), while all other groups in both sexes showed no significant reduction (Table A9 in Appendix 2). Thirdly, sex affected plasma triglyceride levels at all‐time points, with females, on average, having more than six‐fold higher triglyceride levels than the males (*F*_1,49_ = 1232.53, *p* < .001; Figure 3; Table A10 in Appendix 2). This disparity of plasma triglyceride levels between males and females remained significant after controlling for body size.

### 3.4 Triglyceride levels are positively related to body mass

**FIGURE 4:**
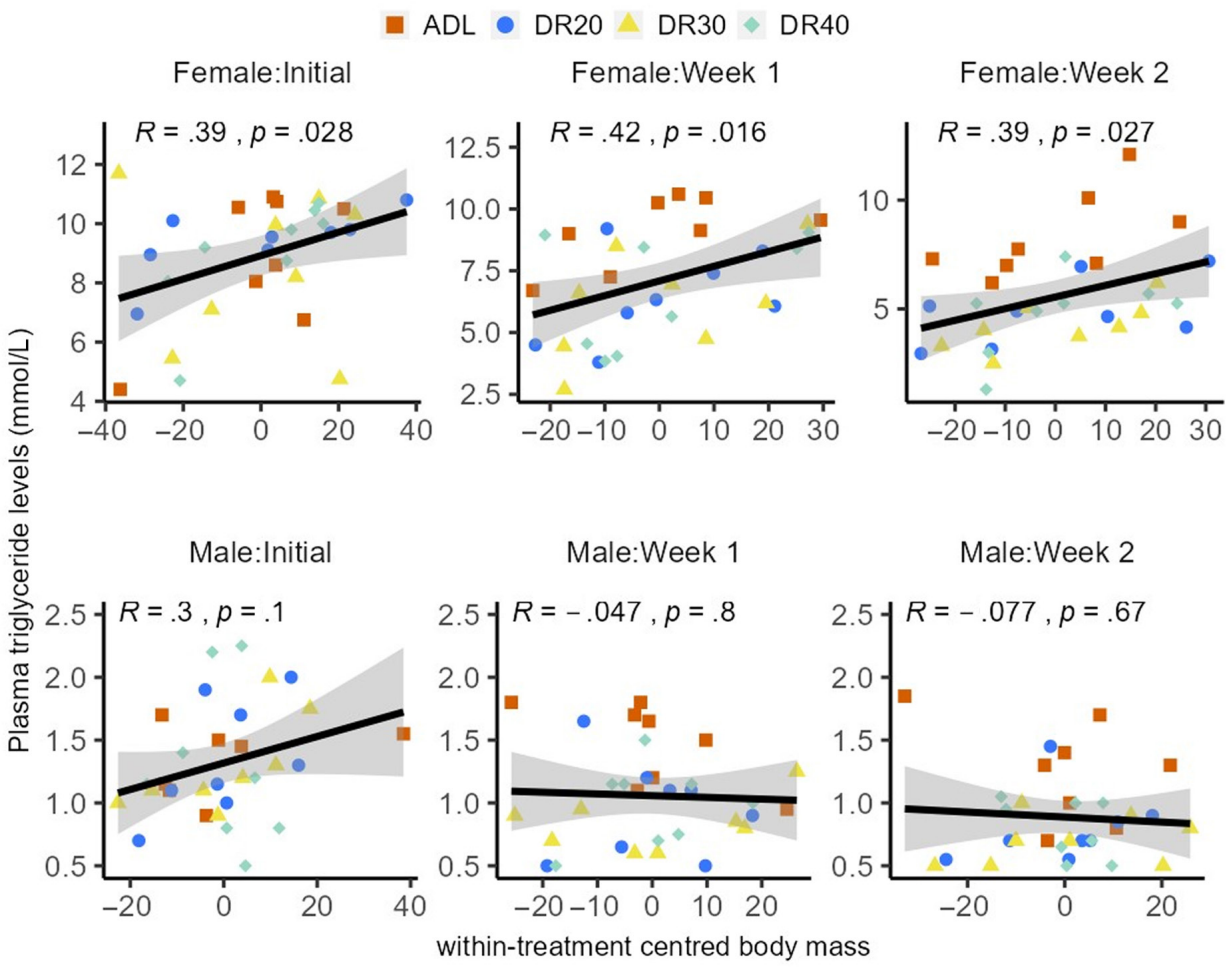
Relationship between body mass and plasma triglyceride levels in females (top panels) and males (bottom panels) at three time points of the experiment. In females, the within‐treatment centred body mass showed a positively significant association with plasma triglyceride levels in females at all‐time points though the association is magnified at week 1 and week 2, while in males there was no association observed. Body mass data was subjected to within‐treatment centring to remove the treatment interference. ADL, ad libitum; DR20, 20% restriction; DR30, 30% restriction; DR40, 40% restriction.

Body mass had significant association with plasma triglyceride levels (Table 1). In females, triglyceride levels showed a significant positive association with body mass at all‐time points, while no significant relationship was observed in males (Figure 4; Figure A6 in Appendix 1). Additionally, while we did not observe significant associations between triglyceride levels and egg mass (Table 2), egg number showed a marginally negative association with plasma triglyceride levels (Table 3). Interestingly, there was also no association between triglyceride and IGF‐1 levels (Figure A6 in Appendix 1), despite that the interaction of triglyceride levels with restriction period (through week 1) significantly explained plasma IGF‐1 (Table 4).

**TABLE 1:** Linear mixed‐effects model for the relationship of body mass with plasma triglyceride levels.

Fixed factors	Estimate	Std. Error	df	*t*‐Value	*p*‐Value^a^	Variance	*R* ^2^
Intercept	−0.333	0.440	122.38	−0.755	.451		
wt_mass	0.008	0.002	124.66	3.496	**.001**		
bt_mass	0.009	0.002	120.57	5.635	**<.001**		
Sex (Male)	−1.614	0.070	71.81	−21.807	**<.001**		
Week 1	−0.121	0.055	156.44	−2.185	**.030**		
Week 2	−0.278	0.060	170.85	−4.606	**<.001**		
wt_mass × sex (Male)	−0.006	0.003	124.75	−1.702	.069		
**Random factor**							
block/birdID						0.028	
block						0.003	
Residual						0.077	
*R* ^2^ marginal							.898
*R* ^2^ conditional							.927

*Note*: *N* = 191. A backward elimination procedure was implemented to find the minimal adequate model from the initial model: lmer(log(triglyceride) ~ wt_mass * sex + bt_mass + week + (1|block/birdID)).

Abbreviations: block/birdID, individual birds identity nested in experimental block; bt_mass, between‐treatment centred body mass; week, restriction period; wt_mass, within‐treatment centred body mass.

^a^ Bold *p*‐values indicate statistical significance.

**TABLE 2:** Linear mixed‐effects model for the relationship of egg mass with plasma triglyceride levels: *lmer(log(triglyceride) ~ wt_eggmass + bt_eggmass + week + (1|block/birdID))*.

Fixed factors	Estimate	Std. Error	df	*t*‐Value	*p*‐Value^a^	Variance	*R* ^2^
Intercept	−13.252	4.427	70.06	−2.993	**.004**		
wt_eggmass	−0.128	0.224	73.46	−0.572	.570		
bt_eggmass	1.813	0.361	69.18	5.028	**<.001**		
Week 1	−0.855	0.413	57.10	−2.071	**.043**		
Week 2	−2.292	0.441	60.98	−5.194	**<.001**		
**Random factors**							
block/birdID						2.302	
block						0.604	
Residual						2.631	
*R* ^2^ marginal							.440
*R* ^2^ conditional							.701

*Note*: *N* = 85.

Abbreviations: bt_eggmass, between‐treatment centred egg mass; wt_eggmass, within‐treatment centred egg mass.

^a^ Bold *p*‐values indicate statistical significance.

**TABLE 3:** Linear mixed‐effect model for the relationship of egg number with plasma triglyceride levels: *lmer(log(triglyceride) ~ wt_egg_number + bt_egg_number + week + (1|block/birdID))*.

Fixed factors	Estimate	Std. Error	df	*t*‐Value	*p*‐Value^a^	Variance	*R* ^2^
Intercept	0.875	0.316	52.97	2.768	**.008**		
wt_egg_number	−0.050	0.029	45.22	−1.718	.093		
bt_egg_number	0.194	0.056	48.44	3.459	**.001**		
Week 2	−0.225	0.069	24.32	−3.260	**.003**		
**Random factors**							
block/birdID						0.047	
block						0.018	
Residual						0.065	
*R* ^2^ marginal							.271
*R* ^2^ conditional							.636

*Note*: *N* = 60. Average egg number for each treatment were ADL, 12.38 ± 0.79; DR20, 10.57 ± 0.81; DR30, 10.00 ± 0.85; DR40, 9.25 ± 0.79.

Abbreviations: bt_egg_number: between‐treatment centred egg number; wt_egg_number: within‐treatment centred egg number.

^a^ Bold *p*‐values indicate statistical significance.

**TABLE 4:** Linear mixed‐effect model for the relationship of plasma triglyceride levels with plasma IGF‐1 levels: lmer(log(IGF‐1) ~ wt_triglyceride * week + bt_triglyceride + sex + (1/birdID)).*eride + sex + (1/birdID))*.

Fixed factors	Estimate	Std. Error	df	*t*‐Value	*p*‐Value^a^	Variance	*R* ^2^
Intercept	2.193	2.168	175.56	11.036	**<.001**		
wt_triglyceride	−0.035	0.027	133.49	−1.340	.183		
bt_triglyceride	−0.019	0.021	132.25	−0.507	.613		
Week 1	0.001	0.049	123.97	0.017	.987		
Week 2	−0.067	0.059	126.32	−1.147	.254		
Sex (Male)	−0.585	0.182	158.65	−3.217	**.002**		
wt_triglyceride × Week 1	0.074	0.033	123.54	2.367	**.019**		
wt_triglyceride × Week 2	0.068	0.039	125.68	1.591	.115		
**Random factor**							
BirdID:block						0.243	
Residual						0.055	
*R* ^2^ marginal							.192
*R* ^2^ conditional							.851

*Note*: *N* = 191.

Abbreviations: bt_triglyceride, between‐treatment centred triglyceride levels; wt_triglyceride, within‐treatment centred triglyceride levels.

^a^ Bold *p*‐values indicate statistical significance.

## 4 DISCUSSION

Birds require adequate and balanced nutrition for various physiological processes. These processes affect their metabolism and fitness components, including growth, reproduction, immune function, feather quality, migration and lifespan (Ben‐Hamo et al., 2017; Blendinger et al., 2022; McWilliams et al., 2004). However, nutritional scarcity is prevalent in nature, resulting in alterations in physiology and subsequently the fitness components (Bonamour et al., 2020). The response to nutritional limitation could be sex‐specific due to differences in reproductive investment and gonadal hormones between males and females (Love et al., 2008; Maklakov et al., 2008). In this study, we used a gradient of dietary restriction (DR) to mimic nutritional limitation and found that (1) DR did not bring about a significant change in IGF‐1 levels; (2) plasma IGF‐1 levels were individually repeatable and higher in females than males; (3) individuals responded differently to dietary conditions; (4) DR revealed a significant reduction in plasma triglyceride levels. However, the effect was not intensified with the severity of restrictions; (5) females had sixfold higher triglyceride levels than males; and (6) plasma triglyceride levels showed a significant positive association with body mass in female quails.

As we have previously reported, DR substantially reduced body mass in both female and male birds, though the effect was more pronounced in females (Reda, Ndunguru, Csernus, Knop, Lugata, et al., 2024; Reda, Ndunguru, Csernus, Knop, Szabó, et al., 2024). Analysis of the weight loss indicated that females experienced significant weight loss at all restriction levels, whereas the loss was milder in males, where only the severe restriction (DR40) proved significant weight loss compared to the control group (Figure 1; Tables A2 and A10 in Appendix 2). The moderate restriction levels did not pose significant weight loss at both week 1 and week 2 of the restriction period (Figure 1). Another study on growing quails also reported a similar trend: females subjected to 15% and 30% restriction for 3 weeks gained less mass than controls, while males were affected only by the 30% restriction (Hassan et al., 2003). Females also lost body mass more than males in broiler chickens subjected to 30% restriction (Tůmová et al., 2022). Male and female birds exhibit different resource allocation strategies driven by their distinct reproductive roles and selective pressures. Females tend to invest more resources in reproduction, while males focus on attracting mates and competing for access to females (Horváthová et al., 2012; Marn et al., 2022). Therefore, the disparate response between males and females to restriction could arise from females having a greater egg production investment, which could be traded off against their body mass. Alternatively, these results may occur due to sexual differences in digestive physiology. Males may experience a longer gut transit time, facilitating effective nutrient absorption to compensate for nutritional deficiencies. Such effects have been documented in sexually dimorphic species (Markman et al., 2006; Santiago‐Quesada et al., 2009), including the Japanese quail (Nóbrega et al., 2020). Longer intestines and higher gut transit times may result in a higher assimilation efficiency in males and they may even possess higher plasticity to grow intestines (Nilaweera & Speakman, 2018; Van Gils et al., 2008) in response to food restriction, although this possibility awaits further research in quails (Table A11 in Appendix 2).

Despite the reducing effect of DR on body mass in our study and on IGF‐1 in human, fly and worm models (ex. Fontana et al., 2008; Kazemi et al., 2020; Regan et al., 2020), we did not observe any directional change in circulating IGF‐1 levels at any restriction levels in our study on adult quails. IGF‐1 is considered to play a crucial role in governing life‐history trade‐offs in growing organisms (Lewin et al., 2017; Regan et al., 2020). Disparities in IGF‐1 concentrations among individuals may result in differences in growth rates and sizes, potentially impacting natural selection and evolutionary processes (Lodjak et al., 2018). However, the effect of IGF‐1 could be variable across species, sex and age of organisms. For instance, a study on bearded reedlings (*Panurus biarmicus*) reported that heavier individuals, who have higher initial circulating IGF‐1, were more likely to decrease IGF‐1 in response to DR than lighter ones (Tóth et al., 2022). However, despite controlling for the effect of individual body mass using within‐individual centring, the treatment's effect on IGF‐1 levels was not affected by either within‐ or between‐individual body mass. Previous reports showed that the levels of circulating IGF‐1 increased with age during early post‐natal development, whereas it gradually reduced after maturity and reached its stable minimum level (Lodjak & Verhulst, 2020; Roberts et al., 1990). Birds develop early‐life insulin resistance, leading to high levels of circulating glucose (Satoh, 2021). This, in turn, inhibits growth hormone secretion, a crucial event for the activation of IGF‐1 secretion. Conversely, the high insulin levels due to insulin resistance stimulate consistent hepatic IGF‐1 secretion at the adult stage (Houston & O'Neill, 1991; Scanes & Braun, 2013). In line with this notion, a recent study at different development stages of chickens revealed that the expression of the IGF system components sharply dropped after hatching and showed no variation between fast and slow growers (Guo et al., 2023). Females also produce local IGF‐1 in the ovary, regulating follicular growth in an autocrine manner without requiring circulating endocrine IGF‐1 (Bernardi et al., 2021; Onagbesan et al., 2009). Therefore, the reaction to nutritional cues in mature birds might also be diminished, which could explain the reduced IGF‐1 response observed across all levels of DR in our study. We also observed large and highly repeatable individual differences in plasma IGF‐1 across time points, consistent with earlier studies in birds and mammals (Lendvai et al., 2020; Obese et al., 2008; Roberts et al., 1990; Tóth et al., 2022), suggesting that differences among individuals could potentially influence how adaptive responses to varying nutritional availability are formed.

Despite the weak response to DR, a distinct difference between females and males was observed: females exhibited 64.7% higher IGF‐1 levels than males (Figure 2). Our findings are consistent with previous reports across different species, suggesting that IGF‐1 exhibits sex‐specific expression (Ashpole et al., 2017; Bacon et al., 1993; Baéza et al., 2001; Johnson et al., 1990; Meter et al., 2022; Tóth et al., 2022; Yuan et al., 2009). Notably, the larger sex exhibits higher IGF‐1 levels, particularly during growth and maturation. Though the exact mechanisms underlying the sex‐related differences in IGF‐1 levels are complex and multifactorial, reproductive investment, sex hormones and sex‐based genetic variations are suggested as major contributors to the difference (Jørgensen et al., 2005; Kerrigan & Rogol, 1992; Liu et al., 2016). In humans, men and women differ in the regularity of GH secretion due to variations in oestrogen levels, subsequently affecting IGF‐1 production (Friedrich et al., 2010; Span et al., 2000). This difference is associated with variations in longevity between males and females (Ahluwalia et al., 2004; Van Heemst et al., 2005). A study on the Eastern fence lizard (*Sceloporus undulates*), a species with female‐biased sexual size dimorphism, also revealed that expression of hepatic IGF1 is reduced in males with increasing testosterone levels, leading to the larger body size in females than males (Duncan et al., 2020). Contrast, in species where males are larger, such as brown anole lizards (*Anolis sagrei*) (Cox et al., 2017), yellow catfish (*Pelteobagrus fulvidraco*) (Ma et al., 2015) and chicken (Yadav et al., 2023), testosterone induced the expression of *IGF1* gene, which also showed a male‐biased pattern. In Madagascar ground geckos (*Paroedura picta*), ovarian hormones suppress *IGF1* expression in females, resulting in smaller females (Meter et al., 2022). In the latter species, the overexpression of *IGF1* in males occurs well after the rise in circulating testosterone levels during sexual maturation. This implies the stimulatory effect of testosterone on IGF‐1, thereby contributing to the larger body size observed in males. In Japanese quails, males and females grow at a similar rate up until the age of 4 to 5 weeks, but males reach sexual maturity 1–2 weeks earlier than females (Ottinger et al., 2005). Testosterone levels significantly begin to rise at the age of 4 weeks and reach their peak stage at 6 weeks (Ottinger & Brinkley, 1978). During this period, males exhibit a slower growth rate compared to females, who gain weight by increasing adipose tissue and mature slowly over a period of 1 or 2 weeks (Lee et al., 2020; Yang et al., 2013). This indicates that in species characterised by larger females in size dimorphism, elevated testosterone levels after sexual maturation in males may inhibit the expression of the *IGF1* gene, thereby reducing the secretion of IGF‐1 and consequently slowing growth. While testosterone is reported to increase growth hormone secretion, thereby upregulating hepatic IGF‐1 production in humans (Gentili et al., 2002; Gibney et al., 2005) and in lizard species with higher male size (Cox et al., 2017), the mechanism of action of testosterone on sexually dimorphic birds needs further study.

**FIGURE A5:**
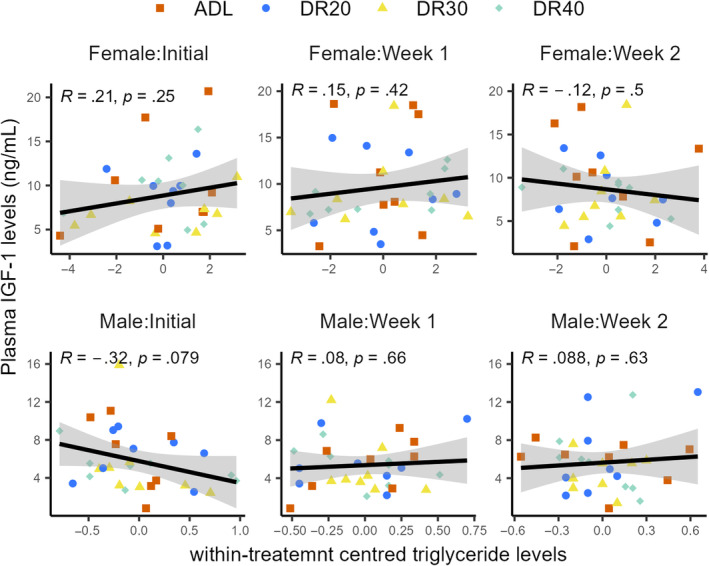
Plasma IGF‐1 and triglyceride levels showed no significant association either in male or female groups, except for initial levels in male groups. ADL, ad libitum; DR20, 20% restriction; DR30, 30% restriction; DR40, 40% restriction.

Furthermore, IGF‐1 levels did not correlate significantly with body mass, egg mass and egg number in our study (Figures A4 and A5 in Appendix 1). A previous comparative study on passerine birds reported that IGF‐1 levels were negatively associated with clutch size and positively associated with egg mass in heavier species (example: *Corvus cornix*, *Garrulus glandarius*, *Turdus* sp.), while the pattern is opposite in species with small body mass (Lodjak et al., 2018). However, the study reported no overall association of clutch size and egg mass with IGF‐1 levels. It has been also reported that *IGF1* increases the expression of genes involved in steroidogenesis in avian preovulatory follicles, which improves egg production (Francoeur et al., 2023). Contrarily, DR during the pre‐laying period increased circulating IGF‐1 levels in domesticated canaries, but did not have a significant effect on egg quality traits (Hargitai et al., 2022).

Our previous report of gene expression patterns from the same experiment also indicated that hepatic *IGF1* gene expression was consistently higher in females than males across dietary regimens (Reda, Ndunguru, Csernus, Knop, Lugata, et al., 2024). Other studies in sexually dimorphic lizards (*Anolis sagrei* and *A. apletophallus*) reported a male‐dominated expression of hepatic IGF‐1 network genes (Cox et al., 2017, 2022). Another study also reported that differences in the expression of IGF‐1 axis genes between sexes contribute to the sexual size dimorphism observed in the yellow catfish (*Pelteobagrus fulvidraco*) (Ma et al., 2015). The studies suggested that sex‐specific expression of the liver *IGF1* gene may play a crucial role in the evolution of species‐specific differences in sexual size dimorphism. However, our finding, in agreement with the study on chicken (Saxena et al., 2020), indicated that the *IGF1* gene was downregulated across restricted feedings, which did not directly translate into changes in circulating IGF‐1 levels in the current study. These findings indicate a potential dissociation between gene expression and protein levels, suggesting that other mechanisms may be involved in regulating circulating IGF‐1. A potential explanation for this discrepancy could be attributed to the metabolic responsiveness of the liver to nutritional fluctuations. Liver genes exhibit an active response to both low or high nutrition (Ahn et al., 2019). In contrast, IGF‐1 can be secreted from other tissues that do not respond rapidly to nutritional changes during the adult stage (Laron, 2001). Furthermore, in another experiment, we also revealed a temporal dissociation between an augmented *IGF1* expression and circulating IGF‐1 levels (Ndunguru et al., 2024). This implies that gene expression patterns may not immediately translate into circulating levels, an effect reported earlier in chickens (Choe et al., 2010).

In this study, we also examined the impact of DR on plasma triglyceride levels. The circulating triglyceride levels have been reduced at all restriction gradients compared to the ad libitum group (Figure 3). Intriguingly, the effect did not exhibit a proportional increase with the severity of restriction. Although there is a lack of previous evidence, this absence of a dose–response relationship could suggest the presence of a threshold effect beyond which further reductions in triglycerides are impeded. This may indicate a potential resource re‐allocation strategy, favouring the maintenance of circulating triglyceride levels at the expense of other traits, which warrants further investigation. While the observed effect trend in males aligned with the previously reported trend in body mass (Reda, Ndunguru, Csernus, Knop, Lugata, et al., 2024), females exhibited a different pattern, wherein the effect on body mass intensified with the severity of restriction (Reda, Ndunguru, Csernus, Knop, Szabó, et al., 2024). The observed difference in sexual trends may be attributed to reproductive investment. Females might compromise their weight to maintain triglyceride levels, ensuring a steady supply for egg production, as de novo triglycerides are mostly deposited into eggs (Cherian, 2015; Vanderkist et al., 2001). In the same report, we also observed a threshold‐like uniform decrease in *IGF1* gene expression (Reda, Ndunguru, Csernus, Knop, Szabó, et al., 2024). Regarding the effect of treatment on triglyceride levels across the restriction period, all restricted groups in both females and males showed a reduced trend. However, the changes were not significant in the second week in both sexes. Previous evidence from different species suggested that dietary restriction can reduce triglyceride levels (Mahoney et al., 2006; Teofilović et al., 2022; Zhan et al., 2007). Triglycerides are mainly derived from dietary sources, particularly from dietary fats and carbohydrates via the liver (Havel et al., 1962). They are prime indicators of fat metabolism (Araújo et al., 2019). In birds, triglycerides serve as energy sources for essential activities such as flight, reproduction and parental care (Fowler & Williams, 2017; Kern et al., 2007). Hence, nutritional limitations forced birds to use body fat reserves and, in the meantime, exposed to a substantial decrease in circulating triglyceride levels (Landys et al., 2005). Furthermore, plasma triglycerides are biomarkers of obesity and cardiovascular disease in humans (Lyu et al., 2022). In broiler chickens, death due to cardiovascular disease is a common cause of loss in farms (Cherian, 2007) and dietary restriction is suggested as a primary solution to prevent such morbidity (Olkowski, 2007).

**FIGURE A6:**
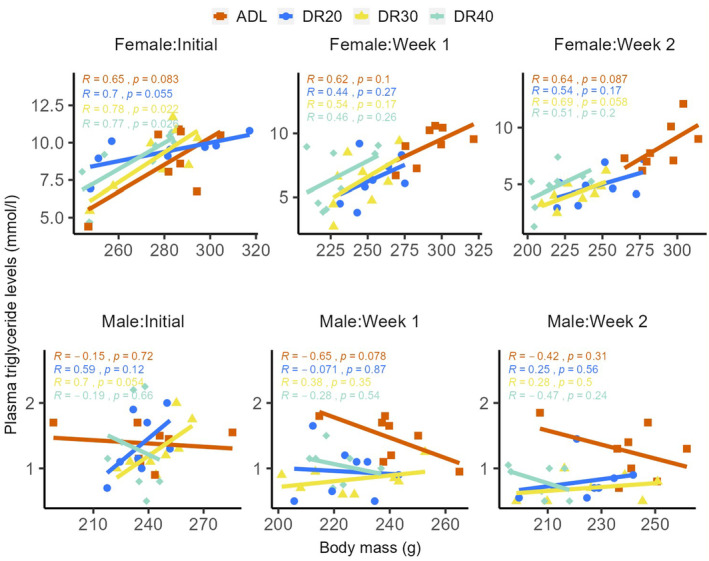
Association of plasma triglycerides to body mass at across treatment levels and time points in female and male Japanese quails. In females, triglyceride levels showed a consistently positive association with body mass in all treatment groups at all time points. In males, the association was inconsistent due to individual differences. ADL, ad libitum; DR20, 20% restriction; DR30, 30% restriction; DR40, 40% restriction.

Female quails exhibited over six‐fold higher circulating triglycerides than males (Figure 3; Table A9 in Appendix 2). These values fall within the reference ranges reported for Japanese quails (Scholtz et al., 2009) and indicate the high hepatic synthesis of fatty acids, cholesterol and phospholipids, which are stimulated by oestrogen and play a critical role in lipid deposition in the yolk of laying hens (Leveille et al., 1957; Salvante et al., 2007; Walzem et al., 1999). Interestingly, in mammals, oestrogen is reported to reduce circulating triglyceride levels via inhibiting feeding behaviour and lipogenesis and promoting triglyceride uptake by adipocytes (Ito et al., 2021). This difference could be attributed to the tendency of females to have higher fat reserves, which is crucial for various fitness components, particularly reproduction, body mass and immunity. We found a positive correlation between plasma triglyceride levels and body mass in females, while no such association existed in males (Figure 4). Within females, heavier individuals had higher triglyceride levels than lighter ones (Figure 4; Figure A6 in Appendix 1). This may be due to the necessity of triglycerides for body fat reserves and their importance in egg deposition for reproductive success. Body fat reserve can have a strong correlation with increasing body mass (Barnett et al., 2015). Interestingly, triglyceride levels had no associations with either egg number or egg mass (Figure A5 in Appendix 1), although triglycerides are used as precursors of egg yolk formation and subsequent egg production (Cui et al., 2020).

## 5 CONCLUSION

Taken together, we found a notable disparity between females and males, with females exhibiting significantly higher IGF‐1 levels compared to males, supporting the notion of sex‐specific IGF‐1 secretion patterns. However, there was no directional response in circulating IGF‐1 levels due to DR. Furthermore, DR reduced plasma triglyceride levels in both females and males. However, females displayed more than a six‐fold higher level than males, suggesting that females require more triglycerides to maintain a circulating energy source and support egg production. Triglyceride levels exhibited a sex‐specific association with body mass, demonstrating a positive relationship in females but no significant relationship in males. This indicates that triglycerides contribute to body fat stores, which, in turn, serve as an indicator of increasing body mass. Overall, our results contribute to the growing need for understanding the sexual differences in avian physiology.

## AUTHOR CONTRIBUTIONS

**Gebrehaweria K. Reda:** Conceptualization (lead); data curation (lead); formal analysis (lead); investigation (lead); methodology (lead); resources (supporting); software (lead); validation (lead); visualization (lead); writing – original draft (lead); writing – review and editing (lead). **Sawadi F. Ndunguru:** Conceptualization (supporting); investigation (supporting); methodology (supporting); writing – review and editing (supporting). **Brigitta Csernus:** Methodology (supporting); writing – review and editing (supporting). **James K. Lugata:** Methodology (supporting); writing – review and editing (supporting). **Renáta Knop:** Methodology (supporting); writing – review and editing (supporting). **Csaba Szabó:** Methodology (supporting); writing – review and editing (supporting). **Levente Czeglédi:** Conceptualization (equal); funding acquisition (lead); investigation (equal); methodology (equal); project administration (lead); resources (equal); supervision (lead); validation (equal); writing – review and editing (equal). **Ádám Z. Lendvai:** Conceptualization (equal); data curation (supporting); funding acquisition (lead); investigation (equal); methodology (equal); project administration (lead); resources (lead); software (supporting); supervision (lead); validation (equal); visualization (supporting); writing – review and editing (equal).

## FUNDING INFORMATION

The study was funded by a grant (K139021) from the National Development, Research and Innovation Fund to Ádám Z. Lendvai and Levente Czeglédi. Gebrehaweria K. Reda, Sawadi F. Ndunguru and James K. Lugata received a Stipendium Hungaricum Scholarship from Tempus Public Foundation for Ph.D. studies. We acknowledge support from the University of Debrecen Program for Scientific Publication.

## CONFLICT OF INTEREST STATEMENT

The authors declare no competing interests.

## Supporting information


Data S1.

## Data Availability

Currently all data and R codes used in this manuscript are attached as Data S1.

## APPENDIX 2 Tables

**TABLE A1:** Ingredient composition and nutrient level of the breeder basal diet.

Feed ingredients	Inclusion rate, %
Corn	30.37
Wheat	20.00
Soybean meal (46% CP)	34.88
Sunflower oil	6.79
Limestone	5.64
MCP	1.29
Salt	0.38
DL‐Methionine	0.15
Vitamin and mineral premix^a^	0.50
Nutrient content, %	
Metabolisable energy MJ/kg	12.13
Crude protein	20.0
Calcium	2.50
Available Phosphorus	0.35
Sodium	0.15
Methionine	0.45
Methionine + cysteine	0.75
Lysine	1.08
Threonine	0.74
Leucine	1.59
Isoleucine	0.86
Arginine	1.33
Tryptophan	0.25

^a^ 1 kg premix provided: 1,000,000 NE vitamin A, 200,000 NE vitamin D_3_, 4900 mg/kg vitamin E, 200 mg vitamin K_3_, 150 mg vitamin B_1_, 500 mg vitamin B_2_, 1200 mg Ca‐d‐Pantothetane, 400 mg vitamin B_6_, 2 mg vitamin B_12_, 11 mg biotin, 2502 mg niacin, 60 mg folic acid, 300,000 mg choline chloride, 13,200 mg Zn, 1920 mg Cu, 9612 mg Fe, 13,200 mg Mn, 180 mg I, 42 mg Se, 12 mg Co.

**TABLE A2:** Model output from a linear mixed‐effects model for mass loss due to dietary restriction across week 1 and week 2 in female and male Japanese quails.

Fixed factors	Estimate	Std. Error	df	*t*‐Value	*p*‐Value	Variance	*R* ^2^
Intercept	9.499	5.075	59.627	1.872	.066		
DR20	−35.603	7.015	51.773	−5.075	<.001		
DR30	−36.478	7.020	51.901	−5.196	<.001		
DR40	−47.191	7.015	51.773	−6.727	<.001		
Week 2	−3.961	2.154	58.000	−1.839	.071		
Sex (Male)	−9.511	6.950	49.925	−1.368	.177		
DR20 × Week 2	−2.719	2.720	58.000	−1.000	.322		
DR30 × Week 2	−6.318	2.765	58.000	−2.285	.026		
DR40 × Week 2	−9.219	2.720	58.000	−3.390	.001		
DR20 × Sex (Male)	25.256	9.733	48.016	2.595	.013		
DR30 × Sex (Male)	23.699	9.911	48.354	2.391	.021		
DR40 × Sex (Male)	30.656	9.733	48.016	3.150	.003		
Week 2 × Sex (Male)	4.658	1.939	58.000	2.415	.019		
**Random factor**							
block/birdID						174.674	
block						7.259	
Residual						29.588	
*R* ^2^ marginal							.57
*R* ^2^ conditional							.94

*Note*: *N* = 128; The final model was *lmer(mass loss ~ treatment * (week + sex) + week * sex + (1|block/birdID))*.

Abbreviations: ADL, ad libitum; DR20, 20% restriction; DR30, 30% restriction; DR40, 40% restriction.

**TABLE A3:** Pairwise comparisons among restriction levels for mass loss across week 1 and week 2 restriction periods in female and male groups.

Sex	Week	Contrast	Estimate^a^	*t*‐ratio	*p*‐value^b^
Female	Week 1	ADL – DR20	34.64	4.84	**<.001**
		ADL – DR30	34.98	4.89	**<.001**
		ADL – DR40	47.46	6.64	**<.001**
		DR20 – DR30	0.34	0.05	.999
		DR20 – DR40	12.83	1.79	.287
		DR30 – DR40	12.49	1.75	.309
	Week 2	ADL – DR20	39.29	5.5	**<.001**
		ADL – DR30	44.3	6.2	**<.001**
		ADL – DR40	56.14	7.85	**<.001**
		DR20 – DR30	5.01	0.7	.896
		DR20 – DR40	16.85	2.36	.097
		DR30 – DR40	11.84	1.66	.356
Male	Week 1	ADL – DR20	11.31	1.58	.396
		ADL – DR30	14.42	1.94	.222
		ADL – DR40	16.26	2.27	.116
		DR20 – DR30	3.11	0.42	.975
		DR20 – DR40	4.95	0.69	.899
		DR30 – DR40	1.84	0.25	.994
	Week 2	ADL – DR20	12.1	1.69	.337
		ADL – DR30	17.46	2.35	.098
		ADL – DR40	26.03	3.64	**.003**
		DR20 – DR30	5.36	0.72	.887
		DR20 – DR40	13.93	1.95	.220
		DR30 – DR40	8.57	1.15	.657

*Note*: The Tukey test was used as a post hoc test with *p* < .05 significance level.

^a^ Std.Error, 7.15; df, 56.

^b^ Bold *p*‐values indicate statistical significance.

**TABLE A4:** Model output from a linear mixed‐effects model for log‐transformed plasma IGF‐1 levels to body mass association. *lmer(log(IGF‐1) ~ wt_mass + bt_mass + sex + (1|birdID))*.

Fixed factors	Estimate	Std. Error	df	*t*‐Value	*p*‐Value^a^	Variance	*R* ^2^
Intercept	1.921	0.364	162.7	5.27	**<.001**		
wt_mass	−0.002	0.002	178.4	−0.74	.460		
bt_mass	0.00001	0.001	149.4	0.49	.626		
Sex (Male)	−0.501	0.132	7.2	−3.80	**<.001**		
**Random factor**							
block**/**birdID						0.23	
Residual						0.06	
*R* ^2^ marginal							.190
*R* ^2^ conditional							.840

*Note*: *N* = 191.

Abbreviations: block/birdID, individual birds identity nested in experimental block; bt_mass, between‐treatment centred body mass; week, restriction period; wt_mass, within‐treatment centred body mass.

^a^ Bold *p*‐values indicate statistical significance.

**TABLE A5:** Model output from a linear mixed‐effects model for plasma IGF‐1 levels to egg mass relationship: *lmer(log(IGF‐1) ~ wt_eggmass + bt_eggmass + week + (1|block/birdID))*.

Fixed factors	Estimate	Std. Error	df	*t*‐Value	*p*‐Value	Variance	*R* ^2^
Intercept	1.486	0.775	75.94	1.92	.059		
wt_eggmass	0.018	0.040	74.78	0.44	.658		
bt_eggmass	0.051	0.062	72.51	0.82	.413		
Week 1	0.0119	0.065	55.92	1.83	.073		
Week 2	0.011	0.070	58.60	0.15	.878		
**Random factor**							
block/birdID						0.105	
block						0.096	
Residual						0.047	
*R* ^2^ marginal							.018
*R* ^2^ conditional							.813

*Note*: *N* = 85.

Abbreviations: bt_eggmass, between‐treatment centred egg mass wt_eggmass, within‐treatment centred egg mass.

**TABLE A6:** Model output from a linear mixed‐effects model for plasma IGF‐1 levels to egg number relationship: *lmer(log (IGF‐1) ~ wt_egg_number + bt_egg_number + week + (1|block/birdID))*.

Fixed factors	Estimate	Std. Error	df	*t*‐Value	*p*‐Value^a^	Variance	*R* ^2^
Intercept	2.233	0.281	43.04	794	**<.001**		
wt_egg_number	0.024	0.022	33.10	1.06	.298		
bt_egg_number	−0.004	0.045	37.00	−0.10	.923		
Week 2	−0.130	0.048	28.48	−2.73	**.011**		
**Random factor**							
block/birdID						0.123	
block						0.094	
Residual						0.030	
*R* ^2^ marginal							.021
*R* ^2^ conditional							.081

*Note*: *N* = 60.

Abbreviations: bt_egg_number, between‐treatment centred egg number; wt_egg_number, within‐treatment centred egg number.

^a^ Bold *p*‐values indicate statistical significance.

**TABLE A7:** Model output from a linear mixed‐effects model for the effect of treatments on plasma triglyceride across time points in female and male Japanese quails.

Fixed factors	Estimate	Std. Error	df	*t*‐Value	*p*‐Value^a^	Variance	*R* ^2^
Intercept	2.162	0.088	87.506	24.569	**<.001**		
DR20	0.021	0.109	154.367	0.197	.8445		
DR30	−0.058	0.109	154.367	−0.533	.5945		
DR40	−0.067	0.109	154.367	−0.609	.5433		
Week 1	0.057	0.098	120.000	0.581	.5622		
Week 2	−0.082	0.098	120.000	−0.831	.4075		
Sex (Male)	−1.887	0.052	52.000	−36.126	**<.001**		
DR20 × Week 1	−0.452	0.139	120.000	−3.245	**.002**		
DR30 × Week 1	−0.445	0.139	120.000	−3.192	**.002**		
DR40 × Week 1	−0.326	0.139	120.000	−2.342	**.021**		
DR20 × Week 2	−0.523	0.139	120.000	−3.757	**<.001**		
DR30 × Week 2	−0.570	0.139	120.000	−4.093	**<.001**		
DR40 × Week 2	−0.478	0.139	120.000	−3.429	**<.001**		
**Random factor**							
block/birdID						0.018	
block						0.008	
Residual						0.078	
*R* ^2^ marginal							.90
*R* ^2^ conditional							.93

*Note* : *N* = 192 ; The final model was *lmer(log(trig) ~ treatment * week + sex + (1|block/birdID))*.

Abbreviations: ADL, ad libitum; DR20, 20% restriction; DR30, 30% restriction; DR40, 40% restriction.

^a^ Bold *p*‐values indicate statistical significance.

**TABLE A8:** Pairwise comparisons of plasma triglyceride levels of all dietary restriction levels at all‐time points in female and male groups.

Sex	Time point	Contrast	Estimate^a^	*t*‐ratio	*p*‐value^b^
Female	Initial (day 0)	ADL – DR20	−0.09	−0.63	.921
		ADL – DR30	0.04	0.26	.994
		ADL – DR40	−0.03	−0.19	.997
		DR20 – DR30	0.13	0.89	.808
		DR20 – DR40	0.06	0.44	.971
		DR30 – DR40	−0.07	−0.45	.969
	Week 1 (day 7)	ADL – DR20	0.37	2.55	**.061**
		ADL – DR30	0.44	2.98	**.020**
		ADL – DR40	0.37	2.49	**.071**
		DR20 – DR30	0.06	0.43	.973
		DR20 – DR40	−0.01	−0.06	.999
		DR30 – DR40	−0.07	−0.49	.961
	Week 2 (day 14)	ADL – DR20	0.55	3.77	**.002**
		ADL – DR30	0.69	4.68	**<.001**
		ADL – DR40	0.64	4.34	**<.001**
		DR20 – DR30	0.13	0.91	.802
		DR20 – DR40	0.08	0.56	.942
		DR30 – DR40	−0.05	−0.34	.986
Male	Initial (day 0)	ADL – DR20	0.05	0.30	.991
		ADL – DR30	0.08	0.46	.966
		ADL – DR40	0.16	0.96	.773
		DR20 – DR30	0.03	0.17	.998
		DR20 – DR40	0.11	0.66	.911
		DR30 – DR40	0.08	0.49	.960
	Week 1 (day 7)	ADL – DR20	0.49	2.67	**.023**
		ADL – DR30	0.57	3.37	**.006**
		ADL – DR40	0.42	2.49	**.068**
		DR20 – DR30	0.08	0.48	.962
		DR20 – DR40	−0.07	−0.40	.978
		DR30 – DR40	−0.15	−0.88	.814
	Week 1 (day 14)	ADL – DR20	0.45	2.67	**.044**
		ADL – DR30	0.57	3.39	**.006**
		ADL – DR40	0.45	2.68	**.043**
		DR20 – DR30	0.11	0.71	.891
		DR20 – DR40	0.001	0.01	1.000
		DR30 – DR40	−0.12	−0.70	.895

*Note*: The Tukey test was used as a post hoc test with *p* < .05 significance level. The final model was *lmer(log(triglyceride)~treatment × week + sex + (1|block/birdID))*. There was a significant treatment × week interaction effect (*p* = .003).

Abbreviations: ADL, ad libitum; DR20, 20% restriction; DR30, 30% restriction; DR40, 40% restriction.

^a^ Female: Std.Error, 0.15; df, 65; Male: Std.Error, 0.17; df, 78.2.

^b^ Bold *p*‐values indicate statistical significance.

**TABLE A9:** Pairwise comparisons of all restriction time points of log triglyceride levels (nmol/L) at each restriction level.

Sex	Treatment	Contrast	Estimate^a^	*t*‐ratio	*p*‐value^b^
Females	ADL	Initial – Week 1	−0.06	−0.44	.890
		Initial – Week 2	0.041	0.31	.947
		Week 1 – Week 2	0.10	0.77	.721
	DR20	Initial – Week 1	0.41	3.09	**.009**
		Initial – Week 2	0.69	5.23	**<.001**
		Week 1 – Week 2	0.28	2.14	**.092**
	DR30	Initial – Week 1	0.34	2.58	**.033**
		Initial – Week 2	0.69	5.24	**<.001**
		Week 1 – Week 2	0.35	2.66	**.027**
	DR40	Initial – Week 1	0.33	2.53	**.037**
		Initial – Week 2	0.71	5.36	**<.001**
		Week 1 – Week 2	0.37	2.83	**.017**
Males	ADL	Initial – Week 1	−0.05	−0.36	.932
		Initial – Week 2	0.12	0.81	.698
		Week 1 – Week 2	0.18	1.17	.476
	DR20	Initial – Week 1	0.38	2.53	**.037**
		Initial – Week 2	0.52	3.45	**.003**
		Week 1 – Week 2	0.14	0.92	.629
	DR30	Initial – Week 1	0.43	2.88	**.015**
		Initial – Week 2	0.61	4.06	**<.001**
		Week 1 – Week 2	0.18	1.18	.470
	DR40	Initial – Week 1	0.20	1.35	.373
		Initial – Week 2	0.41	2.73	**.023**
		Week 1 – Week 2	0.21	1.38	.359

*Note*: The Tukey test was used as post hoc test with *p* < .05 significance level.

Abbreviations: ADL, ad libitum; DR20, 20% restriction; DR30, 30% restriction; DR40, 40% restriction.

^a^ Std.Error, 0.13; df, 56.

^b^ Bold *p*‐values indicate statistical significance.

**TABLE A10:** Pairwise comparisons of females and males for log plasma triglyceride levels at all the treatment levels and time points (week).

Treatment	Week	Contrast	Estimate^a^	*t*‐ratio	*p*‐value^b^
ADL	Initial	Female – Male	1.84	11.63	**<.0001**
	Week 1	Female – Male	1.98	12.54	**<.001**
	Week 2	Female – Male	1.88	11.88	**<.001**
DR20	Initial	Female – Male	2.03	12.83	**<.001**
	Week 1	Female – Male	1.84	11.67	**<.001**
	Week 2	Female – Male	1.95	12.37	**<.001**
DR30	Initial	Female – Male	1.97	12.48	**<.001**
	Week 1	Female – Male	1.90	12.00	**<.001**
	Week 2	Female – Male	1.92	12.14	**<.001**
DR40	Initial	Female – Male	1.81	11.47	**<.001**
	Week 1	Female – Male	1.80	11.39	**<.001**
	Week 2	Female – Male	1.73	10.96	**<.001**

*Note*: The Tukey‐test was used as a post hoc test at *p* < .05 significance level.

Abbreviations: ADL, ad libitum; DR20, 20% restriction; DR30, 30% restriction; DR40, 40% restriction.

^a^ Std.Error, 0.16; df, 144.

^b^ Bold *p*‐values indicate statistical significance.

**TABLE A11:** Mean body mass according to treatment groups, time points and sex.

Sex	Week	Treatment	Mean	SEM
Female	Initial	ADL	283.14	5.93
		DR20	279.69	9.07
		DR30	270.31	7.72
		DR40	268.26	5.97
	Week 1	ADL	292.09	5.83
		DR20	254.00	5.45
		DR30	244.29	6.09
		DR40	229.75	6.22
	Week 2	ADL	289.23	5.75
		DR20	246.49	7.70
		DR30	232.10	5.65
		DR40	218.21	5.30
Male	Initial	ADL	239.89	9.44
		DR20	235.68	4.12
		DR30	245.34	4.90
		DR40	234.25	3.20
	Week 1	ADL	240.43	4.97
		DR20	224.90	4.32
		DR30	226.30	6.49
		DR40	218.53	3.79
	Week 2	ADL	240.05	5.60
		DR20	223.74	4.67
		DR30	225.04	6.54
		DR40	208.39	3.02
