# Supplementary material for: Sex‐specific effects of dietary restriction on physiological variables in Japanese quails
Source: Ecol Evol. 2024 May 23;14(5):e11405. doi: 10.1002/ece3.11405 (PMC11116846; doi:10.1002/ece3.11405)
Supplement: Supplementary file 1 — Data S1. [file ECE3-14-e11405-s001.zip › ECE-2023-11-02071_R_codes.docx]

DR_physiology_R_code

Reda, GK

2024-04-23

# Packeges used

library(tidyverse)
library(lmerTest)
library(readxl)
library(agricolae)
library(emmeans)
library(multcomp)
library(lemon)
library(patchwork)
library(cowplot)
library(grid)
library(gridExtra)
library(rptR)
library(rsq)

# Function to extract legend from a plot

get_only_legend <- function(plot) {
 plot_table <- ggplot_gtable(ggplot_build(plot))
 legend_plot <- which(sapply(plot_table$grobs, function(x) x$name) == "guide-box")
 legend <- plot_table$grobs[[legend_plot]]
 return(legend)
}

# Datasets

# physiology and body mass data
DR_physiology <- read_excel("DR_physiology_dataset.xlsx")%>% group_by(treatment, sex, week)


# within-treatment centering for body mass to physio relationships
wigc <- DR_physiology %>% filter(!is.nan(igf1)) %>% group_by(treatment, sex, week) %>%
 mutate(bt_mass = mean(mass)) %>% # treatment mean of body mass
 mutate(wt_mass = mass-bt_mass) %>% # with in group centered body mass
 mutate(bt_igf1 = mean(igf1)) %>% # treatment mean of igf1
 mutate(wt_igf1 = igf1-bt_igf1) %>% # with in group centered igf1
 mutate(bt_trig = mean(trig)) %>% # treatment mean of triglyceride
 mutate(wt_trig = trig-bt_trig) # with in group centered triglyceride


# egg number and egg mass dataset
DR_egg_data <- read_excel("DR_egg_data.xlsx")%>%
 mutate(eggYesNo = ifelse(is.na(eggmass), 0, 1)) %>%
 #removing birds that did not lay egg before and during the experiment as outliers
 mutate(eggmass = ifelse(birdID == 24, NA, eggmass)) %>%
 mutate(eggmass = ifelse(birdID == 58, NA, eggmass))


# total egg number calculated
egg_number <- DR_egg_data %>% filter(day>0) %>%
 group_by(birdID, treatment) %>%
 dplyr::summarise(n_eggs =sum(ifelse(is.na(eggmass), 0, 1)))

# Summarise and combining egg mass/number data

# summarizing egg mass in to weekly average
egg.mass <- DR_egg_data %>%
 group_by(birdID) %>%
 dplyr::summarize(Initial = mean(eggmass[day == 0], na.rm = TRUE),
 "Week 1" = mean(eggmass[day %in% 1:7], na.rm = TRUE),
 "Week 2" = mean(eggmass[day %in% 8:14], na.rm = TRUE)) %>%
 pivot_longer(cols = Initial:"Week 2", names_to = "week", values_to = "eggmass")


# summarizing egg number into weekly total
eggno <- DR_egg_data %>% filter(day>0) %>% group_by(birdID, treatment) %>%
 dplyr::summarise("Week 1" =sum(ifelse(is.na(eggmass[day %in% 1:7]), 0, 1)),
 "Week 2" =sum(ifelse(is.na(eggmass[day %in% 8:14]), 0, 1))) %>%
 pivot_longer(cols = "Week 1":"Week 2", names_to = "week", values_to = "no_eggs")


# combining physiology and egg data
physio.eggmass <- DR_physiology %>% filter(sex == "Female") %>%
 left_join((dplyr::select(filter(egg.mass, !is.na(eggmass)), birdID, week, eggmass)),
 by = c("birdID", "week")) %>%
 left_join((dplyr::select(filter(eggno, !is.na(no_eggs)), birdID, week, no_eggs)),
 by = c("birdID", "week")) %>%
 mutate(no_eggs = ifelse(birdID == 24, NA, no_eggs)) %>%
 mutate(no_eggs = ifelse(birdID == 58, NA, no_eggs))


# within-treatment centering for egg mass to physio relationships
egg.centering <- physio.eggmass %>% filter(!is.na(eggmass)) %>% group_by(treatment, week) %>%
 mutate(bt_igf1 = mean(igf1)) %>% # treatment mean of igf1
 mutate(wt_igf1 = igf1-bt_igf1) %>% # with in group centered igf1
 mutate(bt_trig = mean(trig)) %>% # treatment mean of triglyceride
 mutate(wt_trig = trig-bt_trig) %>% # with in group centered triglyceride
 mutate(bt_eggmass = mean(eggmass)) %>% #treatment mean of of egg mass
 mutate(wt_eggmass= eggmass-bt_eggmass) # with in group centered egg mass

# within-treatment egg numbers
eggno_centering <- physio.eggmass %>% filter(!is.na(no_eggs)) %>%
 filter(!is.na(igf1)) %>% group_by(treatment, week) %>%
 mutate(bt_egg_number = mean(no_eggs)) %>%
 mutate(wt_egg_number= no_eggs-bt_egg_number)

# Mass loss analyses

# calculating mass loss
masslost <- DR_physiology %>% group_by(sex, block, treatment, week) %>%
 pivot_wider(., id_cols = c(birdID, treatment, block, sex), names_from = "week", values_from = mass) %>%
 rename(week1 = starts_with("Week 1")) %>%
 rename(week2 = starts_with("Week 2")) %>%
 rename(day0 = Initial) %>%
 mutate("Initial" = day0 - day0) %>%
 mutate("Week 1" = week1 - day0) %>%
 mutate("Week 2" = week2 - day0)

# arranging data into longer form
masslost1 <- masslost %>%
 pivot_longer(cols = "Week 1":"Week 2", names_to = "weeklost", values_to = "lostmass")

## Mass loss statistics

# body mass loss final fitted model

masslostmod <- lmer(lostmass ~ treatment * (weeklost + sex) + weeklost * sex + (1|block/birdID), data = masslost1)

# model summary

summary(masslostmod)

# mean comparison

emmeans(masslostmod, specs = pairwise~treatment | weeklost | sex, adjust = "tukey")

## Figure 1. Plotting mass loss due to treatments across time points

# adding initial into the dataset, which is zero to show the intensity of change
masslost2 <- masslost %>%
 pivot_longer(cols = "Initial":"Week 2", names_to = "weeklost", values_to = "lostmass")


#plotting mass lost
masslost2 %>%
ggplot(., aes(weeklost, lostmass, group = treatment, shape = treatment)) +
 stat_summary(fun.data = mean_se, geom = "pointrange",
 position = position_dodge(0.2), size = 0.3, aes(color = treatment, shape=treatment)) +
 stat_summary(fun = mean, geom = "line" ,
 position = position_dodge(0.2), size = 0.5, aes(color = treatment)) +
 facet_rep_grid(.~sex) +
 scale_color_manual(values = c("#d55e00", "black", "#3777FF","#f0e442", "#97D8C4"), name = "") +
 scale_shape_manual(values=c(15, 16, 17, 18), name = "") +
 scale_y_continuous( oob = rescale_none) +
 ylab("Change in body mass (g)") +
 xlab("Restriction period") + labs(color = "") +
 theme(axis.title.y = element_text(size = 8)) +
 theme(axis.title.x = element_text(size = 8)) +
 theme(plot.title = element_text(size = 8)) +
 theme(panel.grid.major = element_blank()) +
 theme(panel.grid.minor = element_blank()) +
 theme(panel.background = element_blank()) +
 theme(panel.border = element_blank()) +
 theme(strip.background = element_blank()) +
 theme(legend.position="non") +
 theme(axis.line = element_line(color = 'black')) +
 # adding statistical letters derived from the mean comparison
 geom_signif(comparisons = list(c("Group1", "Group2")), textsize = 3,
 map_signif_level = TRUE, y_position = 300) +
 geom_text(data = data.frame(weeklost = c("Initial", "Week 1", "Week 2"),
 sex = c("Female", "Male"),
 lostmass = c(0, 10, 14, 0, 14, 8.5, 0, -6.75, -23, 0, -16, -7.1, 0, -16, -29, 0, -19.7, -12.5, 0, -22, -44, 0, -33, -21 ),
 treatment = c("ADL", "DR20", "DR30", "DR40"),
 lab = c("", "a", "a", "", "a", "a", "", "a", "b", "", "b", "ab", "", "a", "b", "", "b", "ab", "", "a", "b", "", "b", "b")),
 mapping = aes(label = lab, color = "black"), show.legend = FALSE, size = 3.5, nudge_x = c(0, -0.04, -0.04, 0, -0.04, -0.04, 0, 0, 0, 0, 0, 0, 0, -0.03, 0.07, 0, 0.1, 0.13, 0, 0.1, 0.14, 0, 0.15, 0.15))

# IGF-1

## Testing effect of handling time, session and staggering on IGF-1 level

handling <- lmer(igf1 ~ handlingtime * treatment + (1|birdID), data = DR_physiology)

summary(handling)


session_effect <- lmer(igf1 ~ session * treatment + (1|birdID), data = DR_physiology)

summary(session_effect)


staggering <- lmer(igf1 ~ stagger * treatment + (1|birdID), data = DR_physiology)

summary(staggering)

## Figure A1. The effect of different dietary restriction levels on levels of plasma IGF-1 at different time points

DR_physiology %>%
ggplot(., aes(week, igf1, group = treatment, shape = treatment)) +
 stat_summary(fun.data = mean_se, geom = "pointrange",
 position = position_dodge(0.2), size = 0.3, aes(color = treatment, shape=treatment)) +
 stat_summary(fun = mean, geom = "line" ,
 position = position_dodge(0.2), size = 0.5, aes(color = treatment)) +
 facet_wrap(~sex) +
 scale_color_manual(values = c("#d55e00", "#3777FF","#f0e442", "#97D8C4"), name = "") +
 scale_shape_manual(values=c(15, 16, 17, 18), name = "") +
 #scale_y_continuous( oob = rescale_none) +
 ylab("Plasma IGF-1 level (ng/mL)") +
 xlab("Experiment periods") + labs(color = "") +
 theme(axis.title.y = element_text(size = 10)) +
 theme(axis.title.x = element_text(size = 10)) +
 theme(plot.title = element_text(size = 10)) +
 theme(panel.grid.major = element_blank()) +
 theme(panel.grid.minor = element_blank()) +
 theme(panel.background = element_blank()) +
 theme(panel.border = element_blank()) +
 theme(strip.background = element_blank()) +
 theme(legend.position="top") +
 theme(axis.line = element_line(color = 'black'))

# Figure 2. Sex difference on IGF-1 levels plot

DR_physiology %>%
ggplot(aes(sex, igf1)) +
 geom_violin() +
 geom_boxplot(width = 0.5, outlier.shape = NA, fill = "white") +
 scale_y_log10() + ylab("Plasma IGF1 levels (ng/mL)") +
 xlab("Sex") + labs(color = "", fill = "") + #geom_jitter() +
 scale_color_manual(values=c("black"), name = "") +
 theme(axis.title.y = element_text(size = 10)) +
 theme(axis.title.x = element_text(size = 10))+
 theme(plot.title = element_text(size = 8)) +
 theme(plot.title = element_text(family = "Times New Roman")) +
 theme(strip.background = element_blank()) +
 theme(panel.background = element_blank())+
 theme(axis.line = element_line(color = 'black'))+
 theme(legend.position = "top") + #ggtitle("(a)") +
 theme(legend.key.size = unit(0.5, 'cm'),
 legend.key.height = unit(0.5, 'cm'),
 legend.key.width = unit(0.5, 'cm'),
 legend.title = element_text(size=8),
 legend.text = element_text(size=8)) +
 # adding asterisks derived from the final model
 geom_signif(comparisons = list(c("Group1", "Group2")), textsize = 3,
 map_signif_level = TRUE, y_position = 300) +
 geom_text(data = data.frame(sex = c("Female", "Male"),
 igf1 = c(20),
 lab = c("***", "")),
 mapping = aes(label = lab, color = "black"), show.legend = FALSE, size = 5, nudge_x = c(0.6, 0))

## Statistical test for IGF-1

# final model
modigf <- lm(igf1~sex, data = DR_physiology)


summary(modigf)

## Figure A2. Individual difference plot for IGF-1

DR_physiology %>% filter(igf1 < 100) %>%
ggplot(., aes(week, igf1, group = birdID, color = treatment, shape = treatment)) +
 geom_point(size = 2) +
 geom_line(size = 1) + facet_wrap(~sex, ncol = 2) +
 scale_y_log10()+
 ylab("plasma IGF-1 level (ng/mL)") + xlab("Experiment periods")+
 scale_color_manual(values=c("#d55e00", "#3777FF","#f0e442", "#97D8C4"), name = "") +
 scale_shape_manual(values = c(15, 16, 17, 18), name = "") +
 theme(legend.position = "top") +
 theme(panel.background = element_blank()) +
 theme(strip.background = element_blank()) +
 theme(axis.line = element_line())

# Individual repeatability test for IGF-1

# Estimate repeatability using lmer

repeatability_model <- lmer(igf1 ~ (1|birdID), data = DR_physiology)

# Extract the variance components
var_components <- VarCorr(repeatability_model)

# Calculate repeatability
repeatability_estimate <- var_components$birdID / (var_components$birdID + attr(var_components, "sc")^2)

# Print the repeatability estimate
cat("Repeatability Estimate:", repeatability_estimate, "\n")

## IGF-1 to body mass association plot with centered value

# males
maleigf1 <- wigc %>% filter(sex == "Male") %>%
ggplot(., aes(wt_mass, igf1)) +
 geom_point(aes(color = treatment, shape = treatment)) +
 facet_wrap(~week, scales = "free",
 labeller = labeller(week = c("Initial" = "Male:Initial", "Week 1" = "Male:Week 1", "Week 2" = "Male:Week 2"))) +
 geom_smooth(method = "lm", aes(color = "black")) +
 stat_cor(aes(label = paste(..r.label.., ..p.label.., sep = "~`,`~")), label.y = 17.5, size = 3) +
 scale_color_manual(values=c( "#d55e00", "black", "#3777FF","#f0e442", "#97D8C4"), name = "") +
 scale_shape_manual(values = c(15, 16, 17, 18), name = "")+
 scale_y_continuous( oob = rescale_none) +
 ylab("Plasma IGF-1 levels (ng/mL)") + xlab("within-treatment centred body mass") +
 theme(axis.title.y = element_text(size = 9, hjust = -1)) +
 theme(axis.title.x = element_text(size = 9))+
 theme(plot.title = element_text(size = 9)) +
 theme(panel.background = element_blank()) +
 theme(panel.border = element_blank()) +
 theme(strip.background = element_blank()) +
 theme(axis.line = element_line(color = 'black')) +
 theme(strip.text = element_text(margin = margin(b = 10))) +
 theme(legend.position = "none") +
 theme(legend.key.size = unit(1, 'cm'),
 legend.key.height = unit(1, 'cm'),
 legend.key.width = unit(1, 'cm'),
 legend.title = element_text(size=12),
 legend.text = element_text(size=12))


# female
femaleigf1 <- wigc %>% filter(sex == "Female") %>%
ggplot(., aes(wt_mass, igf1)) +
 geom_point(aes(color = treatment, shape = treatment)) +
 facet_wrap(~week, scales = "free",
 labeller = labeller(week = c("Initial" = "Female:Initial", "Week 1" = "Female:Week 1", "Week 2" = "Female:Week 2"))) +
 geom_smooth(method = "lm", aes(color = "black")) +
 stat_cor(aes(label = paste(..r.label.., ..p.label.., sep = "~`,`~")), label.y = 23, size = 3) +
 scale_color_manual(values=c( "#d55e00", "black", "#3777FF","#f0e442", "#97D8C4"), name = "") +
 scale_shape_manual(values = c(15, 16, 17, 18), name = "")+
 scale_y_continuous( oob = rescale_none) +
 ylab("Plasma IGF-1 levels (ng/mL)") + xlab("within-treatment centred body mass") +
 theme(axis.title.y = element_blank()) +
 theme(axis.title.x = element_blank())+
 theme(plot.title = element_text(size = 11)) +
 theme(panel.background = element_blank()) +
 theme(panel.border = element_blank()) +
 theme(plot.margin = margin(l = 20, b = 20)) +
 theme(strip.background = element_blank()) +
 theme(strip.text = element_text(margin = margin(b = 10))) +
 theme(axis.line = element_line(color = 'black')) +
 theme(legend.position = "none")

## Figure A3. IGF-1 to body mass association combined panel plot

plot2_legend <- femaleigf1 # inporting the legends from one of the plots

plot2_legend <- plot2_legend +
 theme(legend.position = "top")
legend2 <- get_only_legend(plot2_legend)

combine_plot2 <- grid.arrange(femaleigf1, maleigf1)

igf_mass <- grid.arrange(legend2, combine_plot2, nrow = 2, heights = c(0.7, 10))

## IGF-1 to body mass association statistics

wc_mod_igf <- lmer(log(igf1) ~ wt_mass + bt_mass + sex + (1|birdID), data = wigc)

summary(wc_mod_igf)

## Egg traits to IGF-1 associaiton plot with centered values for female and male

# IGF-1 with egg mass

igf1_eggmass <- egg.centering %>%
ggplot(., aes(wt_eggmass, igf1)) +
 geom_smooth(method = "lm", aes(color = "black")) +
 geom_point(aes(color = treatment, shape = treatment)) +
 facet_wrap(~week, scales = "free",
 labeller = labeller(week = c("Initial" = "(c) Initial", "Week 1" = "(d) Week 1", "Week 2" = "(e) Week 2"))) +
 stat_cor(aes(label = paste(..r.label.., ..p.label.., sep = "~`,`~")), label.y = 20, size = 3) +
 scale_color_manual(values=c("#d55e00", "black", "#3777FF","#f0e442", "#97D8C4"), name = "") +
 scale_shape_manual(values = c(15, 16, 17, 18), name = "")+
 scale_y_continuous( oob = rescale_none) +
 ylab("Plasma IGF-1 levels (ng/mL)") + xlab("within-treatemnt centred egg mass") +
 theme(axis.title.y = element_text(size = 8, hjust = -2.9)) +
 theme(axis.title.x = element_text(size = 8))+
 theme(plot.title = element_text(size = 8)) +
 theme(panel.background = element_blank()) +
 theme(panel.border = element_blank()) +
 theme(strip.placement = "outside", strip.background = element_blank(),
 strip.text.x = element_text(hjust = 0))+
 theme(axis.line = element_line(color = 'black')) +
 theme(legend.position = "none") + #ggtitle("(b)")+
 theme(legend.key.size = unit(0.3, 'cm'),
 legend.key.height = unit(0.3, 'cm'),
 legend.key.width = unit(0.3, 'cm'),
 legend.title = element_text(size=8),
 legend.text = element_text(size=8))

# IGF-1 with egg number

igf1_eggno <- eggno_centering %>% filter(!is.na(no_eggs)) %>%
ggplot(., aes(wt_egg_number, igf1)) +
 geom_smooth(method = "lm", aes(color = "black")) +
 geom_point(aes(color = treatment, shape = treatment)) +
 facet_wrap(~week, scales = "free",
 labeller = labeller(week = c("Week 1" = "(a) Week 1", "Week 2" = "(b) Week 2"))) +
 stat_cor(aes(label = paste(..r.label.., ..p.label.., sep = "~`,`~")), label.y = 20, size = 3) +
 scale_color_manual(values=c("#d55e00", "black", "#3777FF","#f0e442", "#97D8C4"), name = "") +
 scale_shape_manual(values = c(15, 16, 17, 18), name = "")+
 scale_y_continuous( oob = rescale_none) +
 ylab("Plasma IGF-1 levels (ng/mL)") + xlab("within-treatemnt centred egg number") +
 theme(axis.title.y = element_blank()) +
 theme(axis.title.x = element_text(size = 8))+
 theme(plot.title = element_text(size = 8)) +
 theme(panel.background = element_blank()) +
 theme(panel.border = element_blank()) +
 theme(strip.placement = "outside", strip.background = element_blank(),
 strip.text.x = element_text(hjust = 0)) +
 theme(axis.line = element_line(color = 'black')) +
 theme(legend.position = "none") + #ggtitle("(b)")+
 theme(legend.key.size = unit(1, 'cm'),
 legend.key.height = unit(0.7, 'cm'),
 legend.key.width = unit(0.8, 'cm'),
 legend.title = element_text(size=8),
 legend.text = element_text(size=8)) +
 theme(plot.margin = margin(l = 20, r = 100)) +
 theme(legend.key=element_rect(fill="white"))

## Figure A4. IGF-1 to egg traits association combined pannel plot fro female and male

plot2_legend <- igf1_eggno # inporting legend from of the panels

plot2_legend <- plot2_legend +
 theme(legend.position = "top")
legend2 <- get_only_legend(plot2_legend)

combine_plot2 <- grid.arrange(igf1_eggno, igf1_eggmass) # combining the plots

igf_egg <- grid.arrange(legend2, combine_plot2, nrow = 2, heights = c(0.7, 10)) # combining the panel with legend

## Egg mass to IGF-1 association statistical test

wc_igf1_eggmass <- lmer(log(igf1) ~ wt_eggmass + bt_eggmass + week + (1|block/birdID), data = egg.centering)


summary(wc_igf1_eggmass)

## Egg number to IGF-1 association statistical test

wc_igf1_eggno <- lmer(log(igf1) ~ wt_egg_number + bt_egg_number + week + (1|block/birdID), data = eggno_centering)


summary(wc_igf1_eggno)

# Triglyceride

## Testing effect of handling time, session and staggering

# handling
handling_trig <- lmer(trig ~ handlingtime*treatment + (1|birdID), data = DR_physiology)

summary(handling_trig)


# session
session_trig <- lmer(trig ~ session*treatment + (1|birdID), data = DR_physiology)

summary(session_trig)


# staggering

stagger_trig <- lmer(trig ~ stagger*treatment + (1|birdID), data = DR_physiology)

summary(stagger_trig)

## Figure 3. Effetc of dietary restriction on triglyceride levels plot

DR_physiology %>%
ggplot(., aes(week, trig, group = treatment)) +
 facet_wrap(~sex, scales = "free") +
 stat_summary(fun.data = mean_se, geom = "pointrange",
 position = position_dodge(0.2), width = 0.3, size = 0.3,
 aes(color = treatment, shape = treatment)) +
 stat_summary(fun = mean, geom = "line" ,
 position = position_dodge(0.2), size = 0.3, aes(color = treatment)) +
 labs(color = "") +
 scale_color_manual(values=c("#d55e00", "black", "#3777FF", "#f0e442", "#97D8C4"), name = "")+
 scale_shape_manual(values = c(15, 16, 17, 18), name = "") +
 ylab("Plasma triglyceride levels (mmol/l)") + xlab("Experimental periods") +
 scale_y_continuous( oob = rescale_none) +
 theme(plot.title = element_text(hjust = 0.5, size=8)) +
 theme( axis.title.x = element_text(size=8)) +
 theme( axis.title.y = element_text(size=8)) +
 theme(strip.background = element_blank()) +
 theme(axis.line = element_line(color = 'black')) +
 theme(panel.background = element_blank()) +
 theme(legend.position = "none",
 legend.key.size = unit(0.3, 'cm'), #change legend key size
 legend.key.height = unit(0.3, 'cm'), #change legend key height
 legend.key.width = unit(0.3, 'cm'), #change legend key width
 legend.title = element_text(size=8), #change legend title font size
 legend.text = element_text(size=8)) +
 geom_signif(comparisons = list(c("Group1", "Group2")), textsize = 3,
 map_signif_level = TRUE, y_position = 300) +
 geom_text(data = data.frame(week = c("Initial", "Week 1", "Week 2"),
 sex = c("Female", "Male"),
 trig = c(9.85, 1.61, 9.2, 1.52, 9.83, 1.43, 10, 1.12, 5.65, 1.56, 7.3, 0.94, 9.65, 0.72, 3.63, 1.47, 5.1, 0.6, 9.9, 1.14, 5.6, 1.55, 7.65, 0.91 ),
 treatment = c("ADL", "DR20", "DR30", "DR40"),
 lab = c("a", "a", "a", "a", "a", "a", "a", "b", "b", "a", "ab", "b", "a", "b", "b", "a", "b", "b", "a", "ab", "b", "a", "ab", "b")),
 mapping = aes(label = lab, color = "black"), show.legend = FALSE, size = 3.5, nudge_x = c(-0.1, -0.04, -0.04, -0.1, -0.04, -0.04, 0, 0, 0, 0, -0.07, 0, 0.03, 0.04, 0.05, 0.03, 0.05, 0.05, 0.1, 0.17, 0.14, 0.13, 0.12, 0.12))

## Statistical analysis for the effect of dietary restriction, sex and restriction period

trig_lmer <- lmer(log(trig) ~ treatment * week + sex + (1|block/birdID), data = DR_physiology)


summary(trig_lmer)

emmeans(masslostmod, specs = pairwise ~ treatment | weeklost | sex, adjust = "tukey")

emmeans(masslostmod, specs = pairwise ~ weeklost | treatment | sex, adjust = "tukey")

emmeans(masslostmod, specs = pairwise ~ sex | treatment | weeklost, adjust = "tukey")

## Ploting association of triglyceride levels with body mass

# male
maletrig <- wigc %>% filter(sex == "Male") %>%
ggplot(., aes(wt_mass, trig)) +
 geom_point(aes(color = treatment, shape = treatment)) +
 facet_wrap(~week, scales = "free",
 labeller = labeller(week = c("Initial" = "Male:Initial", "Week 1" = "Male:Week 1", "Week 2" = "Male:Week 2"))) +
 geom_smooth(method = "lm", aes(color = "black")) +
 stat_cor(aes(label = paste(..r.label.., ..p.label.., sep = "~`,`~")), size = 3, label.y = 2.5) +
 scale_color_manual(values=c("#d55e00", "black", "#3777FF", "#f0e442", "#97D8C4"), name = "") +
 scale_shape_manual(values = c(15, 16, 17, 18), name = "") +
 scale_y_continuous( oob = rescale_none) +
 ylab("Plasma triglyceride levels (mmol/l)") + xlab("within-treatment centred body mass") +
 theme(axis.title.y = element_text(size = 8, hjust = -0.9)) +
 theme(axis.title.x = element_text(size = 8))+
 theme(plot.title = element_text(size = 8)) +
 theme(panel.background = element_blank()) +
 theme(panel.border = element_blank()) +
 theme(strip.background = element_blank()) +
 theme(strip.text = element_text(margin = margin(b = 10))) +
 theme(axis.line = element_line(color = 'black')) +
 theme(legend.position = "none") +
 theme(legend.key.size = unit(0.8, 'cm'),
 legend.key.height = unit(0.8, 'cm'),
 legend.key.width = unit(0.8, 'cm'),
 legend.title = element_text(size=8),
 legend.text = element_text(size=8))


# female
femaletrig <- wigc %>% filter(sex == "Female") %>%
ggplot(., aes(wt_mass, trig)) +
 geom_point(aes(color = treatment, shape = treatment)) +
 facet_wrap(~week, scales = "free",
 labeller = labeller(week = c("Initial" = "Female:Initial", "Week 1" = "Female:Week 1", "Week 2" = "Female:Week 2"))) +
 geom_smooth(method = "lm", aes(color = "black")) +
 stat_cor(aes(label = paste(..r.label.., ..p.label.., sep = "~`,`~")), size = 3, label.y = 13) +
 scale_color_manual(values=c("#d55e00", "black", "#3777FF", "#f0e442", "#97D8C4"), name = "") +
 scale_shape_manual(values = c(15, 16, 17, 18), name = "")+
 scale_y_continuous( oob = rescale_none) +
 ylab("Plasma triglyceride levels (mmol/l)") + xlab("within-treatment centred body mass") +
 theme(axis.title.y = element_blank()) +
 theme(axis.title.x = element_blank())+
 theme(plot.title = element_text(size = 8)) +
 theme(panel.background = element_blank()) +
 theme(panel.border = element_blank()) +
 theme(plot.margin = margin(l = 18, b = 20)) +
 theme(strip.background = element_blank()) +
 theme(strip.text = element_text(margin = margin(b = 10))) +
 theme(axis.line = element_line(color = 'black')) +
 theme(legend.position = "none") +
 theme(legend.key.size = unit(0.3, 'cm'),
 legend.key.height = unit(0.3, 'cm'),
 legend.key.width = unit(0.3, 'cm'),
 legend.title = element_text(size=8),
 legend.text = element_text(size=8))

## Figure 4. Panel of female and male plots for triglyceride and body mass association

plot2_legend <- femaletrig

plot2_legend <- plot2_legend +
 theme(legend.position = "top")
legend2 <- get_only_legend(plot2_legend)

combine_plot2 <- grid.arrange(femaletrig, maletrig)


trig_masstreat <- grid.arrange(legend2, combine_plot2, nrow = 2, heights = c(0.7, 10))

## Plotting association of triglyceride levels with body mass across all treatment levels

# male
maletrigtreat <- DR_physiology %>% filter(sex == "Male") %>%
 mutate(trig = ifelse(birdID == 9 & week == "Week 1", NA, trig)) %>%
 ggplot(., aes(mass, trig, group= treatment, color = treatment)) +
 geom_point(aes(color = treatment, shape = treatment, )) +
 facet_wrap(~week, scales = "free", labeller = labeller(week = c("Initial" = "Male:Initial", "Week 1" = "Male:Week 1", "Week 2" = "Male:Week 2"))) +
 geom_smooth(se = F, method = "lm", aes(color = treatment)) +
 stat_cor(aes(label = paste(..r.label.., ..p.label.., sep = "~`,`~")), size = 2, label.y = c(2.7, 2.5, 2.3, 2.1)) +
 scale_color_manual(values=c("#d55e00", "#3777FF", "#f0e442", "#97D8C4"), name = "") +
 scale_shape_manual(values = c(15, 16, 17, 18), name = "") +
 scale_y_continuous( oob = rescale_none) +
 ylab("Plasma triglyceride levels (mmol/l)") + xlab("Body mass (g)") +
 theme(axis.title.y = element_text(size = 8, hjust = -0.9)) +
 theme(axis.title.x = element_text(size = 8))+
 theme(plot.title = element_text(size = 8)) +
 theme(panel.background = element_blank()) +
 theme(panel.border = element_blank()) +
 theme(strip.background = element_blank()) +
 theme(strip.text = element_text(margin = margin(b = 10))) +
 theme(axis.line = element_line(color = 'black')) +
 theme(legend.position = "none") + #ggtitle("(b)")+
 theme(legend.key.size = unit(0.8, 'cm'), #change legend key size
 legend.key.height = unit(0.8, 'cm'), #change legend key height
 legend.key.width = unit(0.8, 'cm'), #change legend key width
 legend.title = element_text(size=8), #change legend title font size
 legend.text = element_text(size=8))


# female
femaletrigtreat <- DR_physiology %>% filter(sex == "Female") %>%
 mutate(trig = ifelse(birdID == 14 & week == "Initial", 8.5, trig)) %>%
 mutate(mass = ifelse(birdID == 55 & week == "Initial", 283.7, mass)) %>%
ggplot(., aes(mass, trig, group = treatment, color = treatment)) +
 geom_point(aes(color = treatment, shape = treatment)) +
 facet_wrap(~week, scales = "free", labeller = labeller(week = c("Initial" = "Female:Initial", "Week 1" = "Female:Week 1", "Week 2" = "Female:Week 2"))) +
 geom_smooth(se = F, method = "lm", aes(color = treatment)) +
 stat_cor(aes(label = paste(..r.label.., ..p.label.., sep = "~`,`~")), size = 2, label.y = c(14.2, 13.2, 12.2, 11.2)) +
 scale_color_manual(values=c("#d55e00", "#3777FF", "#f0e442", "#97D8C4"), name = "") +
 scale_shape_manual(values = c(15, 16, 17, 18), name = "")+
 scale_y_continuous( oob = rescale_none) +
 ylab("Plasma triglyceride levels (mmol/l)") + xlab("Body mass (g)") +
 theme(axis.title.y = element_blank()) +
 theme(axis.title.x = element_blank())+
 theme(plot.title = element_text(size = 8)) +
 theme(panel.background = element_blank()) +
 theme(panel.border = element_blank()) +
 theme(plot.margin = margin(l = 18, b = 20)) +
 theme(strip.background = element_blank()) +
 theme(strip.text = element_text(margin = margin(b = 10))) +
 theme(axis.line = element_line(color = 'black')) +
 theme(legend.position = "none") + #ggtitle("(b)")
 theme(legend.key.size = unit(0.3, 'cm'), #change legend key size
 legend.key.height = unit(0.3, 'cm'), #change legend key height
 legend.key.width = unit(0.3, 'cm'), #change legend key width
 legend.title = element_text(size=8), #change legend title font size
 legend.text = element_text(size=8))

## Figure A6. Panel plot for association of triglyceride levels with body mass across all treatment levels

plot2_legend <- femaletrigtreat

plot2_legend <- plot2_legend +
 theme(legend.position = "top")
legend2 <- get_only_legend(plot2_legend)

combine_plot2 <- grid.arrange(femaletrigtreat, maletrigtreat)


trig_masstreat <- grid.arrange(legend2, combine_plot2, nrow = 2, heights = c(0.7, 10))

## Statistics for the association of triglyceride levels with body mass

# fitted model
wc_mod_trig <- lmer(log(trig) ~ wt_mass * sex + bt_mass + week + (1|block/birdID), data = wigc)

summary(wc_mod_trig)

## Statistics for the association of triglyceride levels with egg mass

# fitted model
wc_trig_eggmass <- lmer((trig) ~ wt_eggmass + bt_eggmass + week + (1|block/birdID), data = egg.centering)

summary(wc_trig_eggmass)

## Statistics for the association of triglyceride levels with Egg number

# fitted model
wc_trig_eggno <- lmer(log(trig) ~ wt_egg_number + bt_egg_number + week + (1|block/birdID), data = eggno_centering) # fitted


summary(wc_trig_eggno)

# Plotting IGF-1 to triglyceride levels association

# male
maleigf1_trig <- wigc %>% filter(sex== "Male") %>%
ggplot(., aes(wt_trig, igf1)) +
 geom_point(aes(color = treatment, shape = treatment)) +
 facet_wrap(~week, scales = "free",
 labeller = labeller(week = c("Initial" = "Male:Initial", "Week 1" = "Male:Week 1", "Week 2" = "Male:Week 2"))) +
 geom_smooth(method = "lm", aes(color = "black")) +
 stat_cor(aes(label = paste(..r.label.., ..p.label.., sep = "~`,`~")), label.y = 16.5, size = 2) +
 scale_color_manual(values=c("#d55e00", "black", "#3777FF", "#f0e442", "#97D8C4"), name = "") +
 scale_shape_manual(values = c(15, 16, 17, 18), name = "") +
 scale_y_continuous( oob = rescale_none) +
 ylab("Plasma IGF-1 levels (ng/mL)") + xlab("within-treatemnt centred triglyceride levels") +
 theme(text = element_text(size = 8)) +
 theme(axis.title.y = element_text(size = 8, hjust = -3)) +
 theme(axis.title.x = element_text(size = 8))+
 theme(plot.title = element_text(size = 8)) +
 theme(panel.background = element_blank()) +
 theme(panel.border = element_blank()) +
 theme(strip.background = element_blank()) +
 theme(strip.text = element_text(margin = margin(b = 10), size = 7)) +
 theme(axis.line = element_line(color = 'black')) +
 theme(legend.position = "none") + #ggtitle("(b)")+
 theme(legend.key.size = unit(0.3, 'cm'), #change legend key size
 legend.key.height = unit(0.3, 'cm'), #change legend key height
 legend.key.width = unit(0.3, 'cm'), #change legend key width
 legend.title = element_text(size=8), #change legend title font size
 legend.text = element_text(size=8))


# female
femaleigf1_trig <-wigc %>% filter(sex== "Female") %>%
ggplot(., aes(wt_trig, igf1)) +
 geom_point(aes(color = treatment, shape = treatment)) +
 facet_wrap(~week, scales = "free",
 labeller = labeller(week = c("Initial" = "Female:Initial", "Week 1" = "Female:Week 1", "Week 2" = "Female:Week 2"))) +
 geom_smooth(method = "lm", aes(color= "black")) +
 stat_cor(aes(label = paste(..r.label.., ..p.label.., sep = "~`,`~")), label.y = 20, size = 2) +
 scale_color_manual(values=c("#d55e00", "black", "#3777FF", "#f0e442", "#97D8C4"), name = "") +
 scale_shape_manual(values = c(15, 16, 17, 18), name = "") +
 scale_y_continuous( oob = rescale_none) +
 ylab("Plasma IGF-1 levels (ng/mL)") + xlab("within-treatemnt centred triglyceride levels") +
 theme(text = element_text(size = 8)) +
 theme(axis.title.y = element_blank()) +
 theme(axis.title.x = element_blank())+
 theme(plot.title = element_text(size = 9)) +
 theme(panel.background = element_blank()) +
 theme(panel.border = element_blank()) +
 theme(plot.margin = margin(l = 18, b = 10, t = 10, r = 5)) +
 theme(strip.background = element_blank()) +
 theme(strip.text = element_text(margin = margin(b = 10), size = 7)) +
 theme(axis.line = element_line(color = 'black')) +
 theme(legend.position = "none") + #ggtitle("(b)")+
 theme(legend.key.size = unit(0.3, 'cm'), #change legend key size
 legend.key.height = unit(0.3, 'cm'), #change legend key height
 legend.key.width = unit(0.3, 'cm'), #change legend key width
 legend.title = element_text(size=8), #change legend title font size
 legend.text = element_text(size=8))

## Figure A5. Panel plot fro IGF-1 to triglyceride levels association

plot2_legend <- femaleigf1_trig

plot2_legend <- plot2_legend +
 theme(legend.position = "top")
legend2 <- get_only_legend(plot2_legend)

combine_plot2 <- grid.arrange(femaleigf1_trig, maleigf1_trig)

igf_trig <- grid.arrange(legend2, combine_plot2, nrow = 2, heights = c(0.5, 10))

# Statistics for IGF-1 to triglyceride levels association

# fitted model

igf_trig <- lmer(log(igf1)~wt_trig * week + bt_trig + sex + wt_mass + (1|birdID), data = wigc)


summary(igf_trig)
